# Transcriptome and Proteome Dynamics of a Light-Dark Synchronized Bacterial Cell Cycle

**DOI:** 10.1371/journal.pone.0043432

**Published:** 2012-08-29

**Authors:** Jacob R. Waldbauer, Sébastien Rodrigue, Maureen L. Coleman, Sallie W. Chisholm

**Affiliations:** 1 Department of Civil and Environmental Engineering, Massachusetts Institute of Technology, Cambridge, Massachusetts, United States of America; 2 Joint Program in Chemical Oceanography, Woods Hole Oceanographic Institution and Massachusetts Institute of Technology, Cambridge Massachusetts, United States of America; 3 Department of Biology, Massachusetts Institute of Technology, Cambridge Massachusetts, United States of America; University of Connecticut, United States of America

## Abstract

**Background:**

Growth of the ocean's most abundant primary producer, the cyanobacterium *Prochlorococcus*, is tightly synchronized to the natural 24-hour light-dark cycle. We sought to quantify the relationship between transcriptome and proteome dynamics that underlie this obligate photoautotroph's highly choreographed response to the daily oscillation in energy supply.

**Methodology/Principal Findings:**

Using RNA-sequencing transcriptomics and mass spectrometry-based quantitative proteomics, we measured timecourses of paired mRNA-protein abundances for 312 genes every 2 hours over a light-dark cycle. These temporal expression patterns reveal strong oscillations in transcript abundance that are broadly damped at the protein level, with mRNA levels varying on average 2.3 times more than the corresponding protein. The single strongest observed protein-level oscillation is in a ribonucleotide reductase, which may reflect a defense strategy against phage infection. The peak in abundance of most proteins also lags that of their transcript by 2–8 hours, and the two are completely antiphase for some genes. While abundant antisense RNA was detected, it apparently does not account for the observed divergences between expression levels. The redirection of flux through central carbon metabolism from daytime carbon fixation to nighttime respiration is associated with quite small changes in relative enzyme abundances.

**Conclusions/Significance:**

Our results indicate that expression responses to periodic stimuli that are common in natural ecosystems (such as the diel cycle) can diverge significantly between the mRNA and protein levels. Protein expression patterns that are distinct from those of cognate mRNA have implications for the interpretation of transcriptome and metatranscriptome data in terms of cellular metabolism and its biogeochemical impact.

## Introduction

The relationship between gene expression at the transcript and protein level has broad significance for understanding of cellular function. While the topology of information flow from DNA genes to mRNA transcripts to protein enzymes is established in the central dogma of molecular biology [1], much remains to be resolved regarding how much and how quickly changes in gene product abundance at one level affect downstream abundances and activities. Even in bacteria, in which an mRNA molecule can be simultaneously transcribed and translated, post-transcriptional regulation is increasingly recognized as an important mode of control on the abundances of gene products [2], [3]. Relatively weak correlations between the magnitudes of mRNA and protein abundance changes following laboratory-imposed perturbations have been observed in a number of bacteria, including in responses to IPTG addition [4] and oxygen deprivation [5] in *Escherichia coli*, antibiotic treatment in *Streptomyces coelicor* [6], iron starvation in *Pelagibacter ubique* [7], and carbon starvation in *Caulobacter crescentus* [8]. Such results raise the question of how expression dynamics are coordinated between transcript and protein levels in response to various types of stimuli.

Experiments designed to explore the cascading dynamics of gene expression have typically relied on abrupt, artificially severe stressors to induce expression responses, rather than the natural environmental variability to which organisms have adapted over evolutionary timescales. These types of experiments, while informative, are not likely to reveal the coordination of cellular processes that has been honed by selection in an organism's natural habitat. Multilevel gene-product dynamics have, for the most part, not been explored in cell populations whose growth cycles have been synchronized, either artificially or by entrainment to a natural environmental cue. To date, only one paired transcriptome-proteome analysis of a bacterial cell cycle has been published, that of the unusual asymmetric division of *Caulobacter crescentus* [9]. Expression dynamics observed in unsynchonized populations represent an average of responses of cells across different stages of their cell cycles, which blurs signals that are cell-cycle dependent and may obscure some important operational features of the cellular system.

Here we present paired transcript- and protein-level expression dynamics in a minimal phototroph, the marine cyanobacterium *Prochlorococcus* [10], which is not subject to such limitations. *Prochlorococcus* is naturally synchronized by the diel light-dark cycle in the lab [11], [12] and in the ocean [13], usually doubling once per day on a natural photoperiod (though see [14]). The cells undergo substantial physiological shifts over the course of the diel cycle, with various portions of metabolism dominating at different times of day. Production of biomass via photosynthesis occurs from sunrise throughout the light period, and DNA is replicated in the late afternoon. Cell division begins around sunset and is complete soon after midnight, and at night the cells respire some of the carbon they fixed and stored during the day to maintain ATP and NAD(P)H levels. This synchronization results in well-defined B-, C- and D-phases roughly equivalent to the G1, S and G2+M phases, respectively, of eukaryotic cells [15]. Genome-wide transcriptome analyses over the course of the diel cycle have revealed highly choreographed transcriptional responses to the daily oscillation in energy availability [16]. The naturally induced transcriptional dynamics of the *Prochlorococcus* cell cycle – driven by the daily pulse of energy from light – serve as a useful framework for the analysis of coupled downstream effects presented here.

The Zinser et al. [16] diel study tracked genome-wide transcript-level expression with 2-hour resolution over two successive diel periods in *Prochlorococcus* MED4. They found that 82% of transcripts of protein-coding genes had detectable expression oscillations over the course of the light-dark cycle, which suggests that the diel cycle is the central control on *Prochlorococcus* gene expression under natural conditions. In other cyanobacteria where diel transcriptome oscillations have been measured, smaller proportions of transcripts have been found to cycle with 24-hour periodicity, including 9% in *Synechocystis* [17], 30% in *Cyanothece* [18], 47% in *Crocosphaera watsonii* [19], 25% in *Microcystis aeruginosa* [20], and 32–64% in *Synechococcus elongatus* [21], [22]. The higher proportion of diel-cycling mRNAs in *Prochlorococcus* is likely partially due to the entrainment of the cell cycle to the light-dark cycle and cells dividing once per day, such that all cell-cycle related genes (such as those for DNA replication) are also on a diel cycle. The data reveal a transcriptional program underlying the temporal division of different parts of metabolism: cells are ‘born’ in the middle of the night, and expression of photosynthetic genes peaks around sunrise, priming the cell for carbon fixation and biomass accumulation during the light period. Following completion of chromosome replication around sunset, the direction of carbon metabolism switches as respiratory gene expression peaks, providing energy and reducing power for dark metabolism and cell division, which produces a new daughter cell during the night, and the cycle repeats [16].

A key unresolved question for the *Prochlorococcus* system is the extent to which light-dark induced oscillations in gene expression at the transcript level are actually manifested at the protein level. Are the gene-product abundance variations stronger or weaker at the protein level compared to the respective mRNAs? Is the timing the same – that is, does the abundance of an enzyme wax and wane synchronously with its transcript? If these dynamics are substantially different, this divergence needs to be accounted for in systems-level models of cellular function. Diel transcriptome-proteome analysis of *Cyanothece* has indicated that protein-level oscillations can diverge from those of mRNA [23], [24], but how general these patterns are across phototrophic bacteria remains unclear. In order to draw ecological and biogeochemical inferences from high-throughput metatranscriptomic data obtained from natural environments (e.g., [25], [26]), we need a clearer sense of how mRNA-level variation relates to protein abundance change in a diversity of ecologically-relevant organisms. The diel cycle is one of the strongest yet most predictable perturbations imposed on natural ecosystems, and evolution has selected for strong choreography between this signal and both metabolism and growth in *Prochlorococcus*. Given its enormous global population (estimated at ∼10^27^ cells or 120×10^12^ g of carbon [27]), and the ∼5% of global photosynthesis performed by this single organism (based on daily turnover and the global estimates of gross primary productivity in [28]), understanding how gene expression coordinates with daily variations in light availability has global biogeochemical significance.

## Results and Discussion

### Cell cycle and periodicity of gene transcription and translation

**Figure 1:**
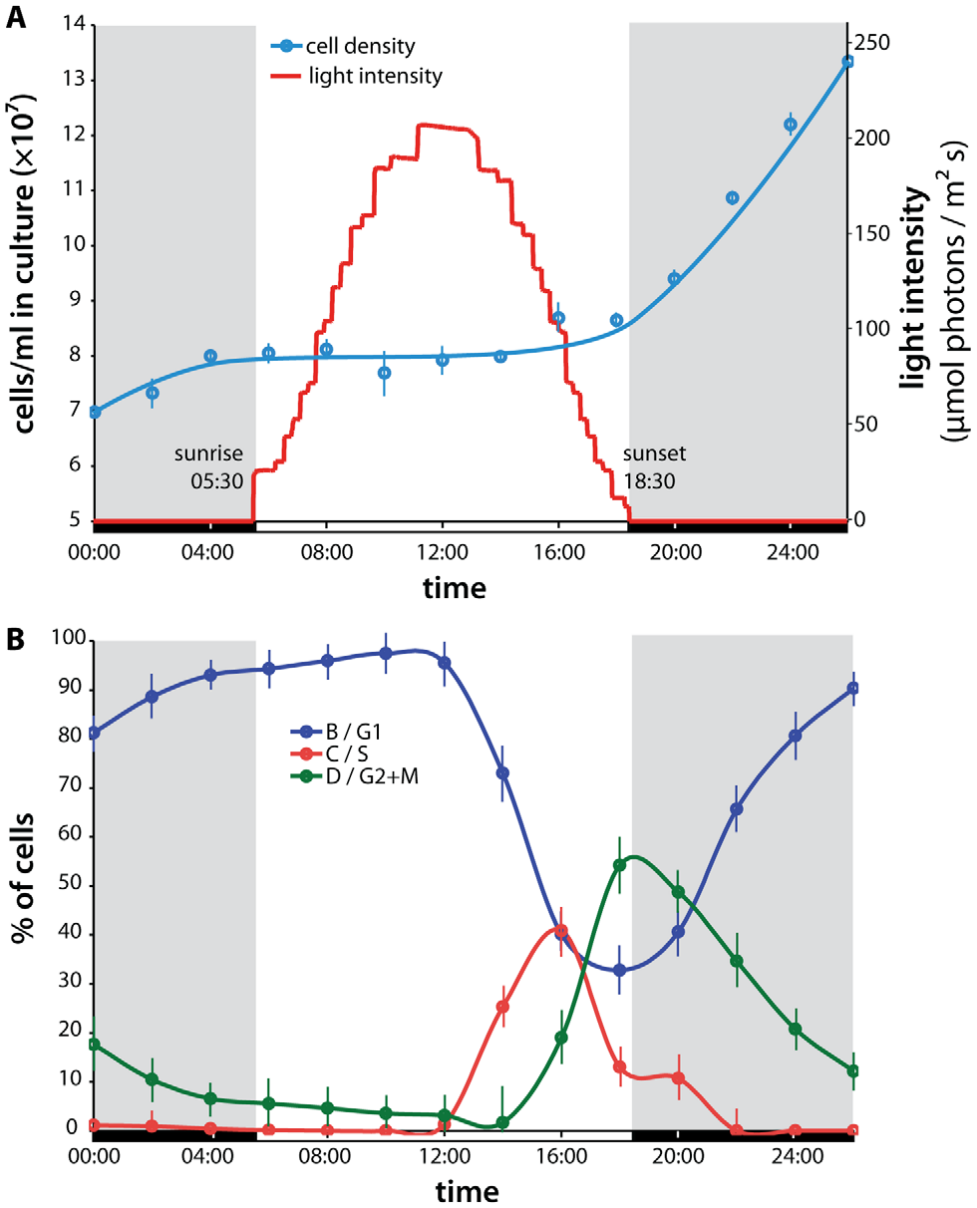
Diel cell growth and cycling. **A** Growth of the *Prochlorococcus* MED4 culture over the diel light/dark cycle. Cell density nearly doubled over the course of the experiment, indicating that 95% of the cells had divided. Local sunrise was at 0530h, and the light period lasted until sunset at 1830h. The simulated natural irradiance curve of the incubator was based on data from irradiance measurements at the Hawaii Ocean Time-series station ALOHA. Sampling for transcript and protein abundance measurements was performed every 2 hours over a 26-hour period. **B** Proportion of cells in different cell cycle phases over the diel period, as determined by DNA staining and flow cytometry. Cells are in B/G1 phase in the predawn through midday, then chromosome replication (C/S phase) begins, peaking just before sunset. Cell division (D/G2+M phase) begins around sunset and is mostly complete by midnight.

*Prochlorococcus* MED4 cultures were synchronized to a diel light-dark cycle with an irradiance curve that simulated natural diurnal conditions (Fig. 1A), resulting in distinct progression through the phases of the cell cycle (Fig. 1B). Total length of the light period was 13 hours, and irradiance peaked at local noon at an intensity of 206 µmol photons/m^2^s. 95% of the cells divided during the period of the experiment. The progression and timing of the cell cycle is consistent with previous experimental observations under laboratory conditions [16] as well as in the ocean [13], and reflects the coherent population behavior that allows us to interpret the population properties as those of an ‘average’ individual cell. Using RNA sequencing (RNAseq) transcriptomics and mass-spectrometry (MS) based proteomics with ^15^N metabolic isotope labeling for quantification (see Methods), we captured the expression dynamics of 1685 transcripts and 548 proteins over the diel cycle with 2-hour resolution (Table 1). When these data were fit to sinusoids with 24-hour periodicity, we detected diel cycling in 1279 mRNA and 312 protein timecourses (all cycling proteins also oscillate at the transcript level). For these 312 genes, we determined both phase (i.e., time of peak abundance) and amplitude (i.e., magnitude of abundance change) of oscillation (Table S2); these are the key parameters we used to compare temporal expression patterns between mRNA and protein. Based on false-discovery-rate analysis that takes into account the sensitivity and specificity of our measurements, we estimate the total proportion of genes with significant diel expression oscillation to be 87% (1464/1685) at the transcript level and 71% (391/548) at the protein level (Table 1 and Table S1); the former result is in agreement with the microarray-based results of Zinser et al. [16].

**Table 1:** Summary of the transcriptome and proteome diel timecourse datasets.

	Genes in Dataset	
	Transcriptome	Proteome
Gene products detected in ≥1 time point	1852	1021
Resolvable abundance timecourses	1685	548
Estimated number of cycling timecourses	1464	391
Timecourses for which phase and amplitude could be calculated	1279	312

The *Prochlorococcus* MED4 genome has 1955 predicted protein-coding genes. Construction of an abundance timecourse for a gene product required sufficient data (generally ≥8 timepoints) after quality filtering (see Methods). The estimated number of significantly cycling timecourses is higher than the number for which specific cycling parameters (phase and amplitude) could be calculated because the former incorporates calculations of the sensitivity and specificity of the cycling analysis (see Methods and Table S1).

**Figure 2:**
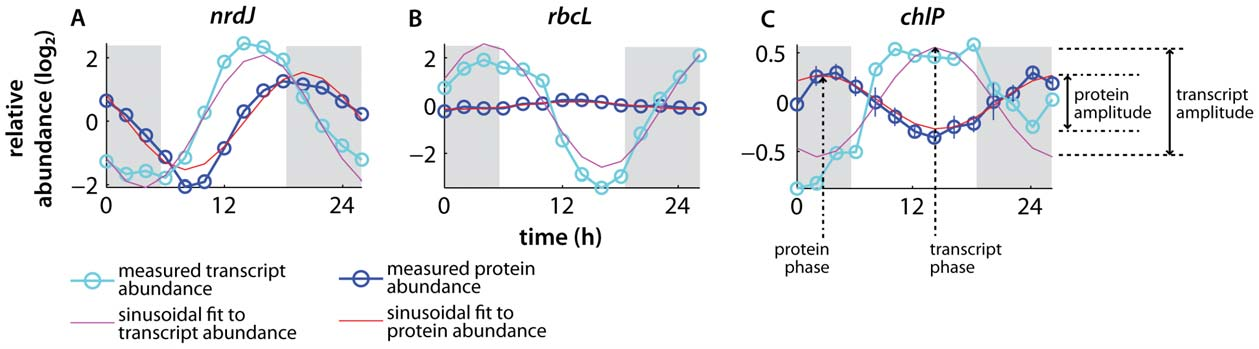
Example protein-transcript relationships. Paired mRNA-protein timecourses over the diel cell cycle, illustrating the variety of relationships seen between transcripts and proteins. Gene product abundances are plotted on a log_2_-transformed scale (i.e., each unit reflects a 2-fold change in abundance). The key parameters of the expression oscillations explored here – the amplitude of oscillation and its temporal phasing – are indicated at right. Where error bars are not shown, they are smaller than the symbol size. **A** Ribonucleotide reductase *nrdJ*, a DNA synthesis enzyme. **B** The large subunit of Rubisco *rbcL*, which fixes CO_2_ into 3-phosphoglycerate in the Calvin cycle. **C** Geranylgeranyl diphosphate reductase *chlP*, a chlorophyll biosynthesis protein. Note that even though the peak of ChlP protein abundance (near 0200h) occurs before that of its mRNA (at 1400h), the protein-transcript lag is still taken to be positive, since the protein maximum is taken to follow the transcript peak from the previous diel cycle.

These 312 paired transcript-protein timecourses reveal a variety of relationships between gene product abundances at the transcript and protein levels, three of which are shown as examples in Fig. 2. Overall, significant divergence between mRNA and protein levels in the relative timing and/or magnitude of abundance oscillations are the rule rather than the exception, and there is a high degree of variability between genes in these transcript-protein relationships. For example, ribonucleotide reductase (*nrdJ*, Fig. 2A), shows strong oscillations of both transcript and protein: the 18.1-fold change in abundance at the mRNA level resulted in a 8.5-fold change in protein abundance, and the peak of expression coincides with C/S-phase just before sunset (Fig. 1), when DNA synthesis is likely most active. By contrast, the magnitude of protein abundance oscillation of *rbcL*, the large subunit of the key carbon-fixation enzyme Rubisco, is more dramatically damped compared to its transcript (Fig. 2B): a 36.8-fold change in transcript abundance results in only a 1.3-fold change in protein level. The abundance of the Rubisco enzyme is thus almost unchanged in *Prochlorococcus* cells between the day when photosynthesis is performed and night when no carbon fixation occurs [16] (discussed further below). The peak of RbcL protein abundance, which occurs in the early afternoon, lags more than 9 hours behind the sunrise maximum of the transcript. Other genes show even more radically phase-shifted behaviors in the relationship between transcript and protein oscillation, including the chlorophyll biosynthesis gene *chlP* for which mRNA and protein oscillations are entirely antiphase (12 hours offset) from one another (Fig. 2C). Given the variety of transcript-protein dynamics observed, we explored a more global, genome-wide view of the relationships between both magnitude and timing of expression oscillations.

### Transcript-protein dynamics: amplitude

**Figure 3:**
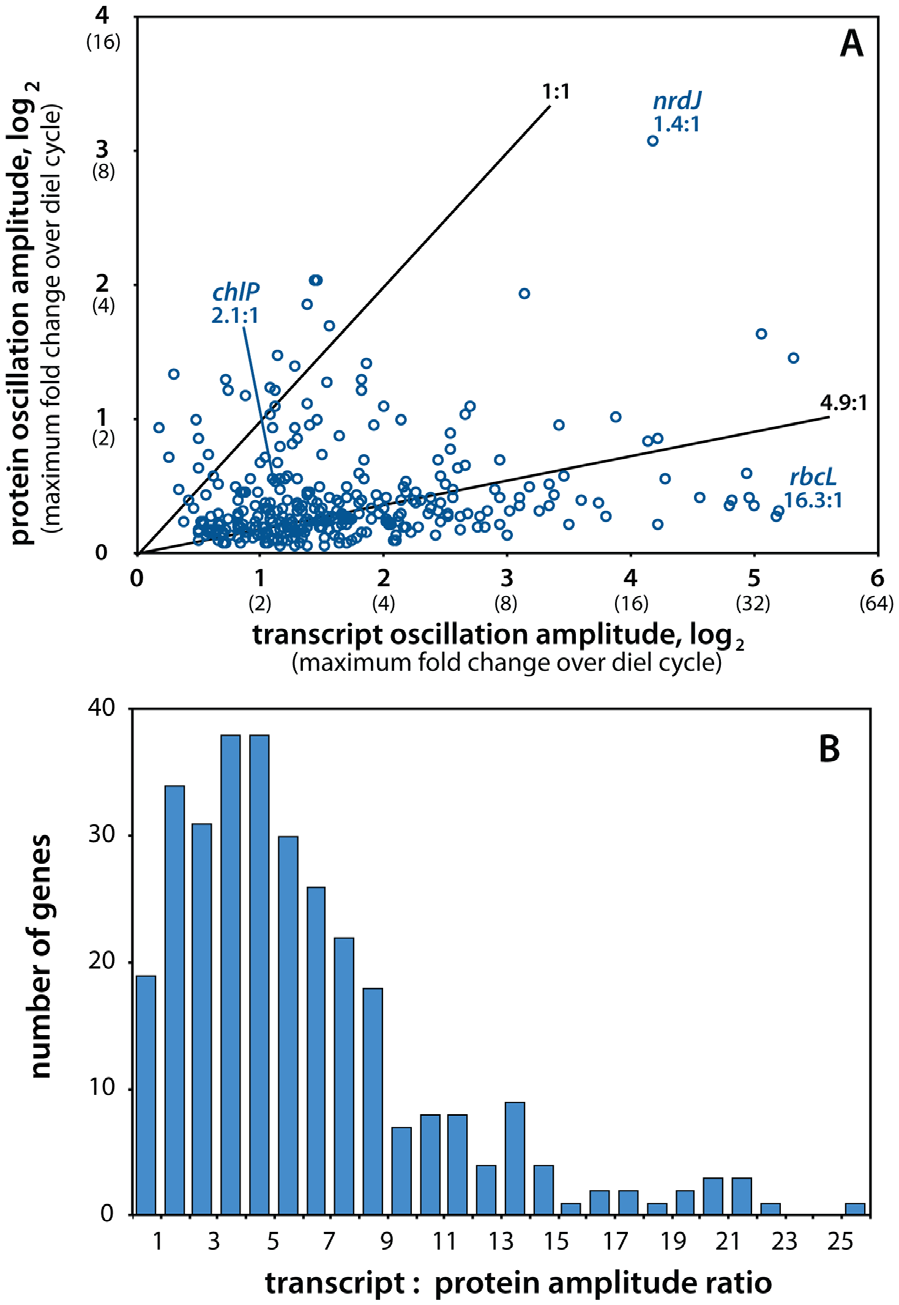
Amplitudes of mRNA and protein oscillations. **A** Comparison of abundance oscillation amplitudes at the transcript and protein levels for the 312 paired expression timecourses. If mRNA and proteins underwent oscillations of the same magnitude, data would plot along the 1∶1 line. The observed median ratio between transcript and protein amplitudes was 4.9∶1 in log2-units, or 2.3-fold greater amplitude at the mRNA level. The three genes shown in Fig. 2, and their amplitude ratios, are indicated. **B** Histogram of transcript-protein amplitude ratios for the 312-gene dataset. For 293 genes (94%) the protein amplitude was damped relative to the corresponding transcript (amplitude ratio >1).

For 293 of the 312 genes for which cycling parameters could be estimated at both the transcript and protein levels, the amplitude of oscillation of the RNA time-course was greater than that of the protein time-course (points below the 1∶1 line in Fig. 3A). The median amplitude across the dataset at the mRNA level was 2.3-fold greater than at the protein-level. The *rbcL* gene products (Fig. 2B), while extreme in their very high transcript-to-protein amplitude ratio, are illustrative of this overall pattern of damped protein-level variation. The observation of significant diel cycling in 16% more transcripts (87%) than proteins (71%) is largely due to damping of those mRNA-level oscillations to below detectability at the protein level. We did not observe any specific correlation between transcript-protein amplitude ratios and gene functional categories (Fig. S1A) or with the time at which genes were maximally expressed (Fig. S2). Amplitude ratios also showed no correlation with the half-lives of mRNAs in MED4 as measured by Steglich et al. [29] (Fig. S3A), so transcript stability does not appear to be a determining factor in the transmission of mRNA-level expression variation to the protein level. Given the large diel oscillations in both transcript levels and the metabolic activity of the cells, this muted temporal variation in protein abundance is surprising, and suggests that fluxes through at least some biochemical networks in the cell are quite sensitive to levels of their constituent enzymes, with abundance changes <2-fold over the diel cycle sufficient to drive metabolic oscillation and the cell cycle.

While the abundance of most proteins in *Prochlorococcus* MED4 oscillates relatively little over the diel cycle (Fig. 3), ribonucleotide reductase (NrdJ; Fig. 2A) changes almost 10-fold between morning and late afternoon, substantially more than any other protein in our dataset (noted in Fig. 3A). This variation is certainly coherent with NrdJ's role in DNA synthesis, which occurs between noon and sunset. Yet other cell-cycle-specific proteins do not oscillate nearly so strongly: FtsZ and MreB, both involved in cell division, vary only by 1.3-fold. It may be that the presence of ribonucleotide reductase in the cytosol outside of C/S phase is deleterious; at other times, the main goal of nucleotide synthesis is RNA, and having material diverted to DNA may be substantially counterproductive. But the presence of ribonucleotide reductase homologues in phage genomes and their expression during lytic infection [30] offers another hypothesis: the low abundance of NrdJ outside of the DNA synthesis may be a mode of defense against phage infection. When a bacteriophage infects a host cell, it shuts down translation of the host genome and begins expressing its own using the host's machinery. Thus, to make progeny, a phage is generally dependent on the presence of DNA-synthesis enzymes (notably NrdJ) in the host cell at the time of infection. If infection occurs when NrdJ protein levels are low (as they are in the morning), infection may stall for want of deoxyribonucleotides to copy the phage genome. In this scenario, the presence of ribonucleotide reductase genes in numerous cyanophage genomes [30]–[32] is a strategy adopted by phage to circumvent dependence on host NrdJ by encoding their own copy and expressing it during infection. The large diel amplitude of *nrdJ* at the protein level could be a symptom of this ongoing ‘arms race’ between phage and host.

### Transcript-protein dynamics: phase

**Figure 4:**
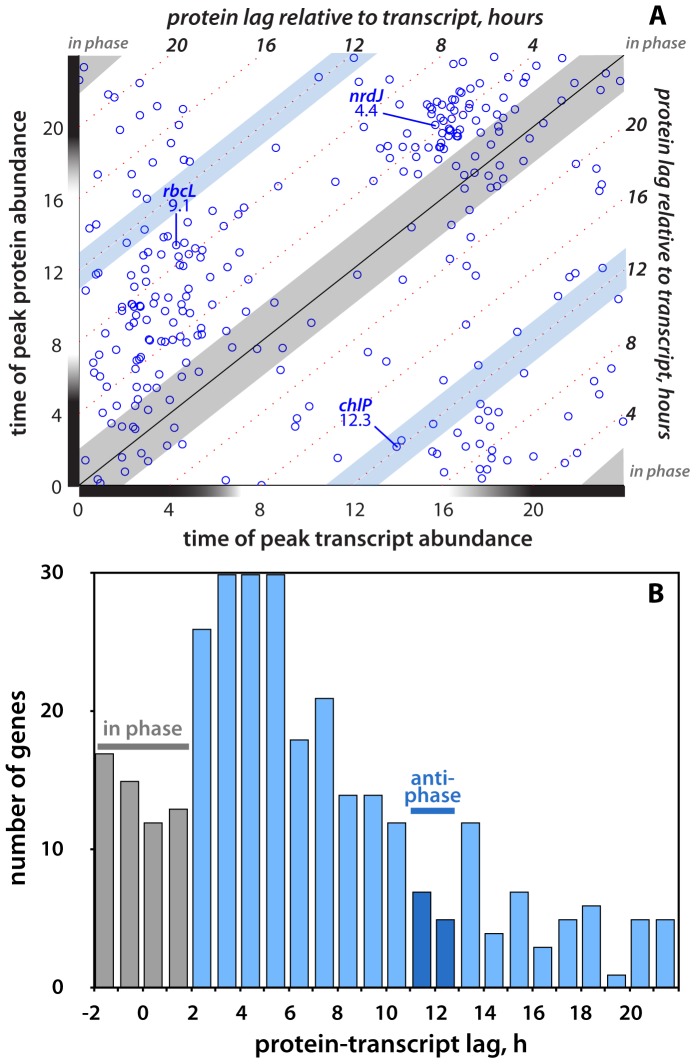
Phasing of mRNA and protein oscillations. **A** Comparison of the phases (i.e., times of peak abundance) of transcripts and proteins for the 312 paired expression timecourses. If oscillations in mRNA and protein abundances were essentially synchronous, most of the data would plot along the main diagonal, within the ±2-hour window that we consider ‘in phase’ based on our sampling resolution. Genes plotting off the in-phase diagonal have progressively longer lag times between protein and trancript oscillations, as indicated by the parallel dotted lines. The three genes shown in Fig. 2, as well as their protein-transcript lag times, are indicated. **B** Histogram of protein-transcript lag times for the 312-gene dataset. Antiphase genes have transcript and protein oscillations offset by close to half of the 24-hour diel cell cycle.

In addition to the relative magnitude of gene expression cycling, we also assessed the phase differences between oscillations of transcript and protein abundance. As reported by Zinser et al. [16], we found that many transcripts are maximally expressed around either sunrise or sunset. When the times of peak abundance of mRNA and protein are compared for the 312 genes, it is apparent that maximal protein abundances generally lag those of transcript by several hours (Fig. 4A). Since our samples were taken every 2 hours, we consider a transcript and protein to be ‘in phase’ if the timing of the peaks of their respective abundance time-courses differ by ≤±2 hours. The distribution of lag times between proteins and transcripts (Fig. 4B) reveals that only 57 of the 312 genes were in phase. For most genes (155/312) the peak of protein expression lagged that of the mRNA by between 2 and 8 hours. For 12 genes (including *chlP*, Fig. 2C), transcript and protein oscillations were completely antiphase – offset by between 11 and 13 hours – meaning that temporal variations in mRNA and protein abundances were essentially anti-correlated. This broad range of temporal offsets between transcript and protein expression oscillations implies that the timing of protein abundance variations is not readily predictable from those of the corresponding mRNA.

As with the transcript∶protein amplitude ratios, neither functional categories (Fig. S1B) nor mRNA half-life (Fig. S3B) showed correlations with protein-mRNA lag times. There is, however, an interesting distinction between the genes whose transcripts peak just before sunrise, between 2am and 5am (*n* = 84), and those that peak near sunset, between 4pm and 8pm (*n* = 79), which together represent about half the protein-mRNA timecourses in our dataset. Evening-peaking transcripts have lower median protein-transcript lag times (4.2 hours) than do morning-peaking transcripts (6.7 hours) and have a tighter distribution of lag times with few very long lags (Fig. S4). We hypothesize that this difference in lag times may result from the timing of cell division, which occurs between sunset and midnight and could prompt cells to translate the cluster of evening-peaking transcripts more rapidly, so that their protein products reach sufficient activity levels to complete cell fission and restart the cycle. The morning-peaking transcripts include a number of photosynthesis and carbon-fixation genes, whose activity is expected to be prolonged throughout the light period, and so the temporal lag between mRNA and protein expression can be extended.

The common protein-transcript lags of 2–8 hours could be explained in part by the low rate of protein production in a small, slow-growing cell like *Prochlorococcus*. While no direct measurements of translation rates in *Prochlorococcus* have yet been made, given estimates of the cellular phosphorus quota [33] and the size of the genome (1.66 Mbp) of this strain of *Prochlorococcus*, we estimate that the cells have only about 600–2,400 ribosomes (Text S1) compared to as many as 70,000 in rapidly-growing *E. coli* cells [34]. Notably, transcript turnover in *Prochlorococcus* is not slower than in other bacteria: the median mRNA half-life is 2.4 minutes, even shorter than in *E. coli* and *B. subtilis* [29], so some transcripts may turn over even before a ribosome is available to translate them. As a consequence, limiting translation could act as a throttle on expression variations in *Prochlorococcus*, both muting and delaying the effect of transcriptional-level regulation on protein abundances for many genes. To explain the observed antiphase mRNA-protein timecourses, however, some form of posttranscriptional regulation is likely required.

### Antisense transcripts

Another possible mechanism to explain the differences between amplitude and phase cycling at the transcript and protein level is the presence of anti-sense RNA molecules. Anti-sense mRNA, transcribed from the DNA strand opposite to the protein-coding gene, can have many effects on gene expression such as altering target RNA stability or inhibiting translation [35]. Translation inhibition results in the presence of transcripts but not of the corresponding proteins, and anti-sense regulation is a plausible explanation for the observed divergences between mRNA and protein abundances. Using a directional RNAseq approach for transcriptome quantification, we detected antisense transcripts for 73.0±6.4% of genes at each time point, and the abundance of antisense RNA was found to be relatively high, with an average of 35.4±5.2% of the corresponding sense message (Table S4). These numbers are substantially higher than the previously reported fraction of antisense RNA molecules in other bacteria [35]. The highest proportion of genes found to have a corresponding anti-sense transcript was 46% in *Helicobacter pylori* [36], and antisense transcription rates in various bacteria ranged between 1.3 and 26.8% [35]. While the abundance of antisense RNA varied during the course of the diel cycle for many genes in our study, reads corresponding to antisense messages showed no preferential location inside sense transcripts in genes where mRNA and proteins were expressed in phase or not (Fig. S5). In addition, genes that show larger differences between protein and mRNA amplitudes did not contain higher antisense transcript levels. Similarly, no distinction in antisense coverage could be detected between genes that are translated in phase or those that displayed phase shifts. Taken together, these observations suggest that neither antisense RNA abundance nor within-gene localization are sufficient to account for the post-transcriptional regulation required to produce at least some the observed differences between transcript and protein expression profiles.

### Diel balance of carbon metabolism in *Prochlorococcus*

**Figure 5:**
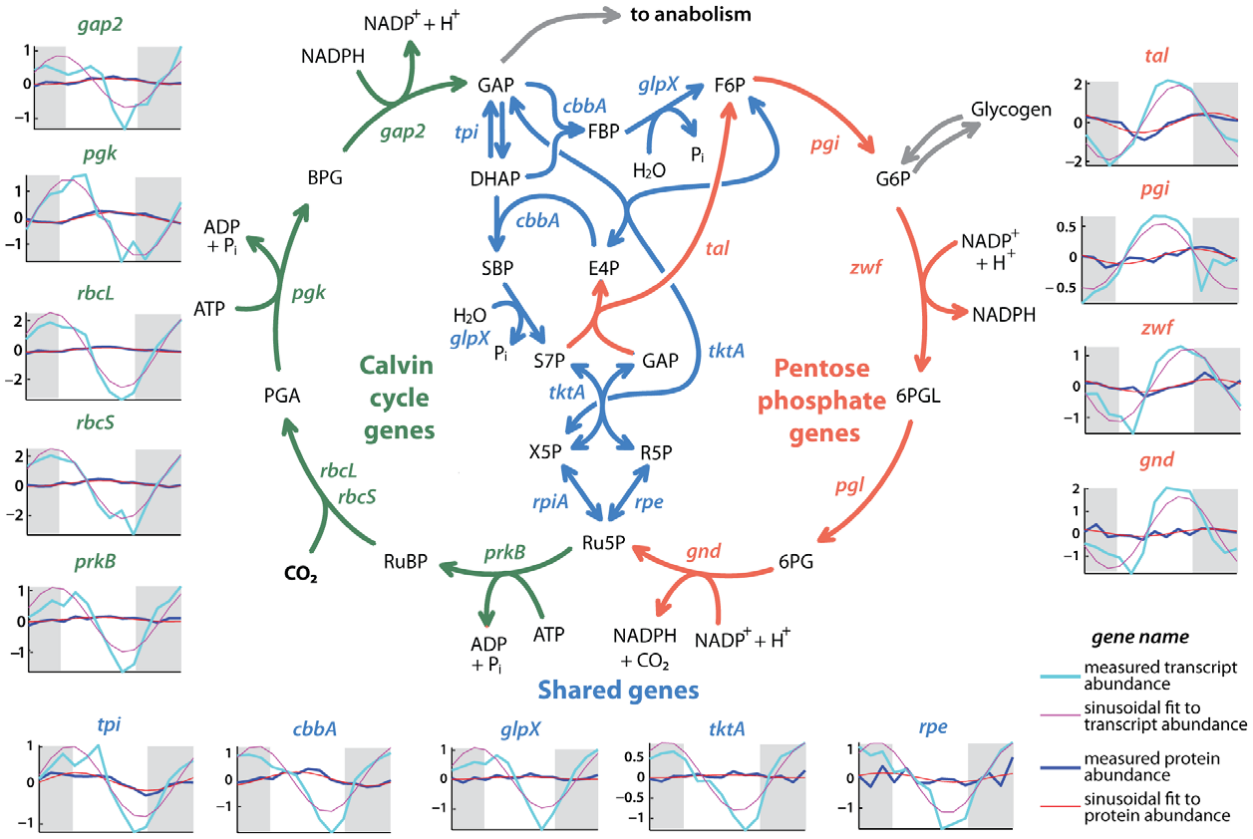
Gene product dynamics of central carbon metabolism. The Calvin cycle-pentose phosphate superpathway of carbon metabolism in *Prochlorococcus*. For each gene, the mRNA and protein timecourse data (and sinusoidal fits to them) are shown. The values of the phase and amplitude of oscillations at both expression levels are given in Table S3. Genes for which timecourses are not shown were either not detected in the proteome or not measurably oscillating in our experiment; note that *cbbA*, *glpX* and *tktA* catalyze multiple reactions. The Calvin cycle consumes CO_2_ and trades reducing power for fixed carbon, while the pentose phosphate pathway does the reverse; reactions in the shared intersection reverse direction depending on the net metabolic flux. The Calvin cycle is the dominant pathway in the light period, when photosynthesis supplies NADPH for carbon fixation. Calvin cycle genes peak near dawn at the mRNA level, and near midday at the protein level. The pentose phosphate pathway is dominant at night, when stores of carbon fixed during the day are respired. Transcripts of pentose phosphate pathway genes peak in the late afternoon, and their proteins after sunset. Genes of the shared intersection show cycling parameters akin to those of the Calvin cycle. For all genes of this superpathway, however, the amplitudes of protein abundance oscillation are much smaller than those of the corresponding mRNA, implying that this redirection of the net flow in this superpathway between light and dark periods is controlled by small changes in protein abundance and posttranslational regulation. Pathway schematic redrawn after [30].

One of the key cellular functions in *Prochlorococcus*, and arguably the most important with regard to its role in ocean biogeochemistry, is carbon fixation using reducing power generated by water-splitting photosynthesis. Two components of the metabolic network responsible for carbon fixation, the Calvin cycle and the pentose phosphate pathway, can be viewed as a superpathway of two intersecting cycles working in opposite directions (Fig. 5). The reductive portion (the Calvin cycle) trades energy (ATP) and reducing power (NADPH) for fixed carbon, while the oxidative portion (the pentose phosphate pathway) trades fixed carbon for reducing power. During the day, photosynthesis can replenish the ATP and NADPH consumed by the Calvin cycle, allowing net fixation of carbon. At night, reserves of fixed carbon stored as glycogen are consumed and NADPH is regenerated. Proper regulation of the intersection of these two cycles is essential: if metabolic flux is allowed to run around the outside of the superpathway, then chemistry accomplished in one part of the cycle is undone in another, with no net result except the waste of 3 molecules of ATP. It is the balance of fluxes through the intersection of the reductive and oxidative portions that determines whether net carbon fixation or respiration occurs [16], [30].

Zinser et al. [16] documented large and temporally-coherent oscillations in the transcript-level expression of Calvin cycle and pentose phosphate pathway genes that coincide with the day-night metabolic division between the two. As in that experiment, we observed that transcripts of Calvin cycle genes peak around sunrise, whereas those of the pentose phosphate pathway peak just before sunset, and both pathways have large amplitudes at the mRNA level (median fold change over the diel of 5.3) (Fig. 5). Our combined proteome-transcriptome dataset, however, reveals that these large variations in mRNA abundance result in only modest changes in protein levels, where the median fold change for the superpathway is just 1.2. If these small changes are sufficient to contribute to redirecting the net flux through this central intersection over the diel cycle, this observation suggests an exquisite balance of this key metabolic network. The relatively constant abundance of Calvin cycle enzymes also helps explain a somewhat puzzling observation that, at night, the maximal light-saturated rate of carbon fixation drops to only 2- to 3- fold below its daytime peak (c.f. Figure 3B of [16]). Our proteome measurements show that, despite large-amplitude oscillations at the transcript level and the redirection of flux towards the pentose phosphate pathway, Calvin cycle proteins are not deeply depleted during the dark period.

Posttranslational regulation of the Calvin cycle and pentose phosphate pathway are likely also important for directing metabolic flow through *Prochlorococcus* central carbon metabolism. We detected OpcA, an allosteric effector of the glucose 6-phosphate dehydrogenase Zwf, at the protein level only at night, when its presence would promote flux through the pentose phosphate pathway. Previous work [16], [30] has highlighted the role of the PrkB/Gap2-binding inhibitor CP12 in modulating operation of this superpathway; while the CP12 transcript is maximally expressed at night, we did not detect the small (74 residue) protein product in this experiment, which was likely outside the analytical window of our proteome measurements. Given the quite small variations in protein levels that accompany the redistribution of metabolic flux through *Prochlorococcus* central carbon pathways, such posttranslational factors may be key players in metabolic regulation. Remarkably, the signature ecological role of *Prochlorococcus* – the steady fixation of carbon and cell growth that make it the base of the food web in much of the open ocean – seems to hinge on its ability to balance the relative abundances of its carbon metabolism enzymes to within a few percent and to regulate their activity post-translationally.

### Comparison with diel expression cycling in *Cyanothece*

The relationships between transcript and protein abundance dynamics over the diel cycle have also been investigated in another phototrophic bacterium, *Cyanothece* ATCC 51142 [18], [23], [24]. Like *Prochlorococcus* MED4, *Cyanothece* 51142 is a marine cyanobacterium, but unlike *Prochlorococcus*, it is a benthic strain that fixes N_2_ and produces H_2_ [37]–[39]. The genome of *Cyanothece* 51142 is substantially larger (5.46 Mb) and structurally more complex, comprising two chromosomes (one circular, one linear) and four plasmids [37]; *Cyanothece* cells are also larger (ca. 2–3 µm [40]) than those of *Prochlorococcus* MED4 (∼0.6 µm). The two organisms thus present contrasts in both ecology and cell biology, and comparison of their overall diel expression cycling characteristics reveals a number of differences.

The total proportion of cycling gene products is higher in *Prochlorococcus* than in *Cyanothece* at both mRNA (87% vs. 30%) and protein (71% vs. 20%) levels [18], [23]. In *Prochlorococcus*, all of the cycling proteins also oscillated at the mRNA level, whereas in *Cyanothece*, 28% (71/250) of cycling proteins did not cycle at the transcript level [23]. Some of this discrepancy may be due to the asynchronous, ultradian growth of *Cyanothece* (10–20 h doubling time [40]), which results in a non-diel periodicity of expression for cell cycle-linked genes and blurring of the signal across the population. As a unicellular diazotroph, *Cyanothece* also expresses nitrogenase at night and so needs to maintain low intracellular O_2_ levels in the dark [37]–[39], a physiological requirement not shared by *Prochlorococcus*. Some apparent differences between the datasets may be due to the different methodologies employed, notably for protein quantification (^15^N metabolic labeling in this study, spectral counting in [23], dynamic/partial incorporation of ^13^C,^15^N-Leu for protein synthesis detection in [24]), as well as different criteria for detecting abundance oscillations.

Other features of diel expression dynamics, however, are broadly congruent between *Prochlorococcus* and *Cyanothece*. Applying the same criteria for in-phase (mRNA and protein peaking within ±2 hours of each other) and antiphase (mRNA and protein abundance maxima 11–13 hours apart) cycling to the two datasets reveals similar proportions in each organism: of the observed expression timecourses, 20% (48/246) are in-phase and 6% (14/246) are antiphase in *Cyanothece*, while 18% (57/312) are in-phase and 4% (12/312) are antiphase in *Prochlorococcus*. Like *Prochlorococcus*, *Cyanothece* operates the Calvin cycle during the day and the pentose phosphate pathway at night [23], [37], [38]. And the relatively large number of actively-synthesized proteins whose transcripts do not cycle was suggested to be indicative of post-transcriptional regulation in *Cyanothece* [24]. Overall, the picture of substantial divergence in the timing and amplitude of diel expression oscillations between transcript and protein levels, and wide variability mRNA-protein relationships among different genes and pathways, appears to hold for both *Cyanothece* and *Prochlorococcus*, and may be a general feature of microbial phototroph physiology in natural habitats.

## Conclusions and Future Directions

The diel cell cycle of *Prochlorococcus* offers a unique window into the relationships between transcriptome and proteome expression dynamics in a biological system responding to a powerful, natural environmental cue. Protein abundance oscillations over the diel cycle are substantially damped compared to temporal variation in both ‘input’ mRNA levels and ‘output’ metabolic activity. Protein-level oscillations are also temporally shifted relative to their coding transcripts – in some cases, shifted far enough that protein abundance was actually increasing while mRNA was decreasing, and vice versa. Such amplitude and phase differences are the rule rather the exception: of the 312 genes for which cycling parameters could be determined at both levels, only 43 (14%) showed what might be considered predominantly transcriptional control, being both in-phase (i.e., with peak times less than ±2 hours apart) and oscillating with amplitudes ≤2-fold different between mRNA and protein. Along with comparable data from *Cyanothece* [23], [24], our results demonstrate that not only the magnitude, but also the timing of protein-level expression variation can diverge substantially from those of the precursor mRNA. This divergence between expression levels is characteristic of a natural, unstressed cell cycle program that has evolved in response to the daily pulse of energy provided by the Sun. As metatranscriptomics develops into a key tool for probing the molecular physiology of marine microbial communities [25], [26], [41]–[43], including in the large subtropical gyres where *Prochlorococcus* is the major primary producer, having a clearer, quantitative understanding of how to link transcript-level expression data to community metabolic activities becomes critical. The picture emerging from multilevel expression studies of model systems suggests that some caution is warranted in extrapolating from temporal patterns of transcript abundance to changes in metabolic fluxes and physiological functioning.

The regulatory mechanisms underlying the strong coupling of gene expression to the diel cycle in *Prochlorococcus* are not yet clear. All strains sequenced to date lack the *kaiA* component of the *kaiABC* cyclic-phosphorylation-based circadian clock system [44], [45], which may contribute to the rapid damping of expression oscillations upon a shift to continuous light [12], [46]. The MED4 genome also encodes only a small complement of the regulatory systems typically found in bacteria [47]–[49], including just five sigma factors and approximately 25 putative DNA-binding transcription regulators. Consequently, regulatory RNAs – such as riboswitches, small noncoding RNAs, or antisense RNAs – potentially play a more important role in controlling gene transcription and transcript stability in this genomically streamlined organism compared to other bacteria [29], [50]. Our data does not support a key role for antisense RNA abundance or within-gene localization *per se* in driving the divergent expression dynamics observed between transcripts and proteins. It is possible, however, that alternative transcript-processing mechanisms not observable in our datasets, such as the RNase III-mediated digestion of double-stranded sense-antisense mRNAs recently described in some Gram-positive bacteria [51], may be operative in *Prochlorococcus*, and are targets for future explorations of this minimal regulatory network.

For transcript levels to predict, or at least strongly correlate with, protein abundance presumes that protein levels are primarily controlled by variations in production rate, with degradation remaining more or less constant. It may be the case, however, that regulated degradation is an important factor in many biological systems [52], [53] and that protein abundance dynamics are controlled as much by loss as by production. And because mass-spectrometry based proteomics measures only protein abundance, not enzymatic activity, it is still only an indirect measure of biochemical action. There is clearly a need for further integration of downstream physiological assays – such as metabolomics in the case of anabolic/catabolic pathways, biophysical measurements of photophysiology, and isotopic tracers of nutrient uptake/assimilation – into genome-wide expression studies, in order to produce a more complete picture of how gene expression is manifested as metabolism. The quantitative concordance between levels of biological organization (transcriptome, proteome, metabolome) is likely to be closest in concerted responses to acute stresses, and more divergent when perturbations are weaker or gradual [54]. Our results provide an example of how evolutionary adaptation to natural, periodic stimuli (such as the rising and setting of the sun) can result in a multilayered expression program. Understanding regulatory responses to natural environmental perturbations on the time scale of microbial growth is an important step towards a mechanistic and predictive picture of how microbial metabolism functions, and how it can drive numerous globally-important biogeochemical processes.

## Materials and Methods

### Diel growth experiment

#### Culture conditions

Axenic *Prochlorococcus* MED4 was grown in batch culture in 30L acid-cleaned polycarbonate carboys (Nalgene) in a modified I-66LL illuminated incubator (Percival Scientific). The illumination in the incubator was controlled by custom PID-controlled dimmer circuitry and programmed to match a diel irradiance curve measured at the Hawaii Ocean Time-series station ALOHA (Fig. 1). Temperature in the incubator was maintained at 24°C. Each culture was stirred continuously by a large teflon-coated magnetic stir bar. Prior to inoculation of the large-volume batch cultures for this experiment, the culture had been maintained for several months in the same incubator to ensure synchronization to the light-dark cycle. The culture medium was a modified version of Pro99 [55] based on Vineyard Sound seawater (collected at Woods Hole, MA) and pH-buffered with 10 mM HEPES and 6 mM sodium bicarbonate. Starting culture volume for the diel growth experiment was 20L. Separately, *Prochlorococcus* MED4 labeled with ^15^N were prepared by growing cells on Pro99 medium in continuous light, with >99% ^15^NH_4_Cl (Cambridge Isotope) as the sole fixed N source. A 1L culture was grown to late-exponential phase and harvested by centrifugation as described below, with samples preserved for flow cytometry to provide accurate cell counts of the pellets.

#### *Sampling*

During the diel growth experiment, samples were taken every two hours over a 26 hour span, beginning at local midnight, resulting in 14 total timepoints. At each timepoint, 500 ml samples of each culture were withdrawn using spigots at the bottom of the carboys into 2×250 ml centrifuge bottles. For sampling during dark and low-light periods, a low-power green lamp was used to provide indirect, non-photosynthetically-active work light. Additionally, five 1 ml samples of the culture were preserved with 0.125% glutaraldehyde (Tousimis) for flow cytometric determination of cell density and growth cycling. Large-volume samples were centrifuged at 16,000×*g* for 10 minutes. After pipetting off the supernatant, the two pellets from each culture were resuspended, combined in a 15 ml conical tube, and the volume brought to 5 ml with Pro99 media. 10 µl aliquots of the resuspended concentrate were diluted 1∶100 with 0.125% glutaraldehyde in Pro99 in for flow cytometric analysis to ensure precise and accurate determination of cell counts in the sample pellets. The remainder of the concentrate was then split into 2×2 ml and 4×0.25 ml aliquots in 2 ml microcentrifuge tubes (Sarstedt), the former for transcriptomics and the latter for proteomics. These aliquots were centrifuged for 6 minutes at 14,000×*g*, and the supernatant was then removed and preserved with 5 µl 25% glutaraldehyde in cryovials for flow cytometric counting to assess pelleting efficiency. Cell pellets and flow cytometry samples were kept frozen at −80°C until analysis.

#### Flow cytometry

Cell counts and cell cycle were analyzed using an InFlux flow cytometer (BD Cytopeia). Glutaraldehyde-fixed samples were diluted in filtered sterile seawater to appropriate concentrations. Light scatter and fluorescence signals were detected using a 488 nm excitation beam, triggering on forward (small-angle) light scatter and counting cells on the basis of scatter characteristics and chlorophyll fluorescence. Cell cycle analysis was performed using SYBR Green (Invitrogen) to stain DNA. Flow cytometry data were analyzed using FlowJo (TreeStar).

### Transcriptomics sample preparation & analysis

#### RNA extraction

Total RNA was extracted from frozen cell pellets with the mirVana kit (Ambion) as previously described [16]. Genomic DNA was removed by digestion with Baseline-ZERO DNase (Epicentre Biotechnologies) and RNA was concentrated and recovered with the RNA Clean & Concentrator-5 kit (Zymo Research) according to the manufacturer's recommendations (general procedure protocol). The RNA was quantified using a Nanodrop spectrophotometer (Thermo).

#### Directional RNA-seq

A strand-specific RNA-seq protocol was developed for analyzing the *Prochlorococcus* transcriptome. 150 ng of total RNA was fragmented by magnesium catalyzed hydrolysis (40 mM Tris-Acetate, pH 8.1, 125 mM KOAc, 37.5 mM MgOAc) for 5 minutes at 95°C, and purified with the RNA Clean & Concentrator-5 kit (Zymo Research) according to the manufacturer's recommendations (general procedure protocol). The resulting RNA molecules were polyadenylated (Ambion) and immediately treated with antarctic phosphatase before inactivation of the enzyme and phosphorylating 5′ RNA extremities with T4 PNK. A DNA-RNA hybrid adaptor (5′-ACACGACGrCrUrCrUrUrCrCrGrArUrCrU-3′) was then ligated to the 5′ end of RNA fragments and purified with RNA clean XP beads (Beckman Coulter genomics) to remove unligated excess adaptor. This RNA was used for reverse transcription reaction using an anchored oligo dT16-VN Illumina adaptor (5′-CTCGGCATTCCTGCTGAACTTTTTTTTTTTTTTTTVN-3′). The cDNA was then amplified by qPCR with Phusion DNA polymerase and the reaction was stopped towards the end of the exponential amplification phase as monitored on an Opticon qPCR instrument (Bio-Rad). The amplified library was finally purified with AMPure XP beads (Beckman Coulter genomics). The resulting library was quantified and the fragment size distribution was determined using a Bioanalyzer 2100 (Agilent).

#### Illumina sequencing

Illumina sequencing was performed on a Genome Analyzer IIx. 36 cycles were performed but split between the actual sequence (30 nt) and an internal 6 nt barcode located inside one of the adaptors. Approximately 7 million reads were obtained for every sample by multiplexing 4 samples per lane.

#### Data analysis

Raw reads were sorted according to their barcodes using Novobarcode (Novocraft). Fastq files were aligned against the *Prochlorococcus* MED4 reference genome using MAQ [56] to generate a pileup file describing coverage in the sense and anti-sense orientation for each bp. Coverage inside genes was summed, divided by gene length, and normalized on total number of bp aligned to the reference genome using custom PERL scripts to allow quantitative comparison of transcript abundance between samples.

#### Affymetrix microarrays

In parallel with RNA-sequencing, at least 100 ng of total RNA was used for amplification with the MessageAmp II-Bacteria kit (Ambion). The resulting cRNA was hybridized to custom Affymetrix MD4-9313 arrays. The data was normalized and analyzed as previously described [16]. See Text S1 for comparison of the diel transcriptome from microarray and RNAseq results (Figure S9 and Figure S10).

### Proteomics sample preparation & analysis

#### Protein extraction & SDS-PAGE

*Prochlorococcus* cell pellets were extracted with LDS buffer (Invitrogen), reduced with dithiothreitol, and cysteine thiols alkylated with iodoacetamide. Extract from sample pellets was then mixed 1∶1 by cell numbers (established previously by flow cytometry) with extract from an identically and simultaneously processed ^15^N-labeled cell pellet. Protein extracts were loaded onto 10% SDS-PAGE gels (NuPAGE, Invitrogen) and separated by electrophoresis with MOPS running buffer. Gels were stained with SimplyBlue coomassie (Invitrogen) and imaged on a flatbed scanner prior to slicing into eight separate molecular weight fractions for each timepoint. SDS-PAGE efficiently separated high molecular weight protein from other components of the cell extract, notably chlorophyll, which is abundant in *Prochlorococcus* cells, and residual salts, all of which could interfere with downstream analytical steps.

#### Trypsin digestion & peptide extraction

Gel pieces were destained, washed, fully dehydrated and chilled on ice. Gel pieces were saturated with sequencing grade modified porcine trypsin (Promega) and incubated for 48 hours at 37°C. Peptides were extracted twice with 10% acetonitrile in 50 mM ammonium bicarbonate alternating with 50% acetonitrile in 0.1% formic acid. Extracts were dried by vacuum centrifugation at 30°C and stored frozen until analysis.

#### Liquid chromatography/mass spectrometry

Dried peptide fractions (8 per timepoint) were resuspended 5% acetonitrile/0.25% formic acid and injected with a MicroAS autosampler (Thermo/Spark Holland). The liquid chromatography system consisted of a Surveyor pump (Thermo) fitted with a backpressure regulator (P-880, Upchurch) and a fixed-T flow splitter (ratio ∼90∶1), and a reversed-phase capillary LC column (Hypersil Gold C18, 0.18×100 mm, 3 µm×175 Å particles, Thermo). The mobile phase system was 0.1% formic acid in water (buffer A) and 0.1% formic acid in acteonitrile (B); peptides were eluted with a gradient of 5% to 37.5% B over 105 minutes at a flow rate of 1.3 µl/min. Nanospray ionization was performed with a TriVersa Nanomate (Advion). Post-column flow splitting produced a flow of ∼400 nl/min to the nanospray chip. The spray chip was operated at a voltage of 1.6–1.8 kV, and spray current was monitored to ensure ionization stability. Mass spectral data was acquired on a LTQ-FT Ultra (Thermo) in a data-dependent manner. Each full scan in the ICR cell (profile mode, m/z 300–1600, resolution 100,000) was followed by 4 CID MS/MS scans on selected precursors in the linear ion trap. The Top4 method was designed to provide good temporal resolution in the MS1 data for the purposes of ^15^N-based quantification. Dynamic exclusion was enabled, with repeat count set to 2 and exclusion duration 30 seconds. Singly charged ions, ions whose charge state could not be assigned, and common contaminant ions were excluded from MS/MS precursor selection.

#### Peptide/protein identification

Peptide-spectrum matching was performed against a database consisting of the *Prochlorococcus* MED4 genome [57], its reversed complement, and a set of common contaminant proteins including porcine trypsin and human keratins. Three MS/MS database search algorithms were employed: X!Tandem ([58]; with the k-score plugin [59]), MyriMatch [60] and OMSSA [61]. For all search engines, semi-tryptic searches were conducted with two missed cleavages allowed. Amino acid modfications included static carbamidomethylated cysteine, variably oxidized methionine, and variable formation of pyro-glutamine, -glutamate or -carbamidomethylcysteine on the N-termini of peptides. Peptide identification probabilities were assigned with PeptideProphet [62] and merged across search engines and gel slices using iProphet [63]. Peptide identifications were then assigned to proteins by ProteinProphet [64] using Occam's razor logic, and requiring a minimum PeptideProphet score of 0.05 to filter out the weakest spectrum IDs. Further filtering based on an arbitrary ProteinProphet score cutoff was not necessary at this point, as the false discovery rate (FDR) was already acceptably low: 33 decoy proteins were identified among 1021 MED4 proteins, for a nominal dataset-wide protein-identification FDR of 3.2%. The number of timepoints at which each protein was detected is shown in Figure S6; in the unfiltered dataset, 360 proteins were detected at all 14 timepoints. As discussed below, the process of constructing expression timecourses eliminated all decoy data, resulting in a protein-ID FDR in the timecourse data of <0.2%.

### Protein quantification & cycling analysis

#### Protein timecourse quantification

To provide a single internal standard that could be used to quantify proteins across the diel cycle, a separate *Prochlorococcus* MED4 culture was grown under continuous-light conditions (i.e., unsynchronized) with ^15^NH_4_ as a sole nitrogen source. Metabolic isotope labeling using ^15^N has a number of advantages for the type of high-precision protein timecourse quantification needed in this experiment. Mixing the samples taken at each diel sampling timepoint with aliquots of ^15^N-labeled cells at the earliest stage of protein extraction minimizes the effect of extraction yields and different protein solubilities on the observed abundance ratio, because those biases apply equally to both sample and standard. Similarly, potential biases due to subsequent sample preparation steps, such as variable enzymatic digestion efficiencies or peptide extraction yields, are experienced equally by the intermixed sample and standard. Accurate cell counts of both the ^14^N diel samples and the ^15^N internal standard pellets by flow cytometry ensured that the ^14^N/^15^N mixing ratio was near 1∶1.

MS1 peaks corresponding to ^15^N-labeled isotopologues of identified MED4 peptides were matched to their unlabeled, coeluting partners by ASAPRatio [65], and the abundance ratios of the ^15^N- versus ^14^N-peaks computed. A window of 0.05 *m/z* was used for integration, multiple charge states were used for quantification, and peakgroup pairs were constrained to the same elution time range. ASAPRatio estimates an integrated intensity error for each peak by the difference between the integrated raw intensity and the integrated area under a fitted peak generated by a Savitzky-Golay smoothing filter; this error was used to calculate a coefficient of variation (CV) for each individual peakgroup ratio. The total dataset comprised isotope ratios for 95,542 unique peakgroup pairs – here referred to as “peaks” for brevity, but actually representing in excess of 500,000 distinct LC-MS features, including both ^14^N- and ^15^N-partners and ^13^C-isotopologues for each peptide across multiple charge states.

The set of unique peak ratios was then filtered to remove peaks with a ratio CV greater than 41%, corresponding to the 80th percentile in CV ranking. These high-CV peaks are generally of low intensity and/or poor peak shape, making them more prone to quantification errors. The remaining data were then log_2_-transformed, and the peak ratios were adjusted to compensate for slight variations away from 1∶1 in the mixing ratio of ^14^N- and ^15^N-protein at different timepoints. This was done by finding the median of the log_2_-transformed peak ratios for each timepoint, and subtracting that value from each peak ratio at that timepoint. This normalization procedure ensures that the peak ratio distributions for each timepoint have a common median (i.e., 0 on a logarithmic scale) and that timecourse variations for indivdual proteins are not due to systematic biases in the overall dataset.

Protein-level expression timecourses were constructed from the peak-level data in the following manner: for a given protein, if four or more peaks were found at eight or more timepoints, those timepoints were included in the timecourse. If a timecourse could not be constructed with those criteria, the threshold number of peaks required for that protein was sequentially lowered (to 3, 2, or 1) until 8 or more timepoints were included. This approach was chosen to maximize the quality of the extracted timecourses for data-rich proteins, but to also allow timecourses to be constructed for proteins consistently detected at low levels. For timepoints with at least 4 peaks, the set of peak ratios was tested for outliers using a variation of the integrated inconsistent rate (IIR) method [66], with an IIR cutoff value of 1.81.

Protein expression ratios were calculated from the peak-level data at each timepoint by maximum likelihood estimation of the parameters of a lognormal fit to the data, taking the mean of the lognormal distribution as the protein ratio. The uncertainty in the ratio was taken as the upper and lower 95% confidence limits calculated from an unbiased estimate of the standard error of the mean [67] given by: ±(1.96*c*N*s*)/(*N* ^0.5^), where *s* is the standard deviation of the lognormal fit to the peak-level data, *N* is the number of (filtered) peaks observed for a given protein at a given time, and the factor *c*N is taken from Table 2 of [67].

Finally, 70 outlier timepoints (1.1% of the 6157 total) were excluded from protein expression timecourses by measuring the ratio difference between each point in a timecourse and its nearest neighbors in time. Timepoints whose summed nearest-neighbor distances were greater than an empirically-determined threshold value of 4.15 times greater than the mean value for that timecourse were identified as outliers. This timepoint outlier detection method does not assume any underlying model for temporal structure in the data. The effect of each of the filtering steps described above on the dataset are summarized in Table S5. The filtered dataset used for timecourse construction included 66,186 peak ratios, or 69.2% of the full dataset before filtering. Ultimately, expression timecourses over the diel cycle were constructed for 548 proteins.

#### Analysis of diel transcript & protein expression oscillation

Analysis of the temporal cycling of transcript and protein expression followed an approach based on that outlined by Futschik and Herzel [68]. A best-fit sinusoid with a 24-hour period was found for each expression timecourse. To gauge the quality of the fit, and hence the strength of the cycling oscillation, Fourier scores were calculated for all timecourses as in Zinser et al. [16] (Fig. S7A). To assess the significance of a given Fourier score value, a background distribution of scores from 1000 simulated first-order autoregressive (AR(1)) timecourses was generated for each gene product, using autoregression coefficients and white noise distributions derived from the timecourses. Futschik and Herzel [68] have shown that an AR(1) background model is a more stringent and specific test of cyclic expression than Gaussian or randomized backgrounds. From comparison with these background distributions, a *p*-value was derived for each gene product for the null hypothesis of no significant 24-hour cycling (Fig. S7B). Multiple hypothesis testing was then performed on each of these two collections of *p*-values (for 1685 transcript timecourses and 548 protein timecourses) with the program QVALUE (v. 1.1; [69]); parameters included use of the the polynomial method for π_0_ and robust *q*-value method. A *q*-value threshold for significant diel expression oscillation was chosen from inspection of the QVALUE results (Fig. S8 and Table S1).

### Data deposition

Transcriptomic and proteomic data from this study are available from the Dryad repository at: http://dx.doi.org/10.5061/dryad.8kk12 as well as from ProPortal at: http://proportal.mit.edu [70].

## Supporting Information

Text S1 **Discussion of estimation of ribosome numbers in** ***Prochlorococcus*** **cells and comparison of transcriptome dynamics as measured by RNA-sequencing and microarrays.**
(PDF)

Figure S1 **Ranges of (A) transcript∶protein oscillation amplitude ratio and (B) lag time for cycling genes grouped by Gene Ontology category.**
(PDF)

Figure S2 **Relationships between the transcript∶protein oscillation amplitude ratio and (A) the time of peak transcript abundance and (B) the time of peak protein abundance.**
(PDF)

Figure S3 **Relationships between the half-lives of transcripts in** ***Prochlorococcus*** **MED4 as determined by Steglich et al.** [**29**] **and (A) transcript∶protein oscillation amplitude ratio and (B) lag time between peaks of transcript and protein expression; neither cycling parameter correlates with mRNA half-life.**
(PDF)

Figure S4 **Distributions of protein-mRNA lag times for genes whose transcripts peak between 2am and 5am (upper panel), just before sunrise, and 4pm and 8pm (lower panel), around sunset.**
(PDF)

Figure S5 **Transcriptome coverage along the length of protein-coding genes.** No clear difference in depth of coverage or localization of either sense or antisense reads was observed between gene expressed in phase or not in phase at the transcript and protein levels.
(PDF)

Figure S6 **Number of timepoints at which each MED4 protein was detected.**
(PDF)

Figure S7 **Distributions of Fourier scores (A and B) and** ***p*** **-values for significant diel cycling (C and D) for the 1685 transcript timecourses (A and C) and 548 protein timecourses (B and D) in the dataset.**
(PDF)

Figure S8 **Results of QVALUE calculations (see Methods) on cycling** ***p*** **-value distributions for transcriptome (A and B) proteome (C and D) timecourses.** Red lines indicate the chosen *q*-value cutoffs.
(PDF)

Figure S9 **Comparison of phases (i.e., times of peak abundance) of transcripts as measured by RNA-sequencing and microarrays.**
(PDF)

Figure S10 **Comparison of amplitudes of transcript abundance oscillations as measured by RNA-sequencing and microarrays.**
(PDF)

Table S1 **Results of QVALUE** [**69**] **analysis of transcriptome and proteome timecourse datasets.**
(PDF)

Table S2 **Cycling parameters of the 312 genes for which the phase and amplitude of diel oscillation could be calculated at both transcript and mRNA levels.**
(PDF)

Table S3 **Cycling parameters of genes of the Calvin cycle (green), pentose phosphate pathway (red) and shared between the two (blue).**
(PDF)

Table S4 **Proportion of genes with detected sense or anti-sense transcript at each experimental timepoint.**
(PDF)

Table S5 **Effect of data-quality filtering steps (see Methods) on proteomics dataset size.**
(PDF)
