# Supplementary material for: Transcriptome and Proteome Dynamics of a Light-Dark Synchronized Bacterial Cell Cycle
Source: PLoS One. 2012 Aug 29;7(8):e43432. doi: 10.1371/journal.pone.0043432 (PMC3430701; doi:10.1371/journal.pone.0043432)
Supplement: Text S1 — Discussion of estimation of ribosome numbers in Prochlorococcus cells and comparison of transcriptome dynamics as measured by RNA-sequencing and microarrays. (PDF) [file pone.0043432.s001.pdf]

Supporting Text

Ribosome numbers in *Prochlorococcus* cells

We can estimate the number of ribosomes in a *Prochlorococcus* cell on the basis of a cellular phosphorus budget. Several studies have presented measurements of the elemental composition of *Prochlorococcus* cells [33,S1,S2] and provide a basis for major-element budgets. These studies indicate a range of cellular P contents between 0.12 and 0.32 amol, with the lower end likely more representative of natural oligotrophic ocean conditions and the higher end of growth in nutrient-replete media.

Phosphorus is present in *Prochlorococcus* as a component of three main classes of biochemicals: nucleic acid polymers (DNA and RNA), phospholipids, and small molecule metabolites. The amount in the chromosome can be calculated exactly from the genome size (1.66Mbp), and here for simplicity we consider cells in the B/G1 phase with a single copy of the chromosome. Phospholipids are rather rare in *Prochlorococcus* compared to most other microbes, likely as an adaptation to chronic P scarcity, and they do not upregulate phospholipid biosynthesis under P-replete conditions [S3]. We calculate the total number of lipid molecules in the cell by assuming a typical geometry for *Prochlorococcus*: the cell envelope (outer membrane, murein layer and inner membrane) is set at 0.6μm in diameter. Within the cell are two complete, spherical thylakoid membranes averaging 0.5μm in diameter [S4] – for geometric simplicity, we neglect the small 'hairpin' region where each membrane wraps around itself to enclose the lumen. Thus the inner and outer cell membranes comprise 4 leaflets (2 bilayers), while the thylakoids comprise 8 leaflets (4 bilayers), and we assume a membrane area of $0.55 nm^2$ per lipid molecule [S5] and that lipids constitute 80% of total membrane area [S6]. Hence our hypothetical cell contains about 14 million lipid molecules, 2% of which are assumed to be phosphatidylglycerol [S3]. Little data exists on metabolites or their concentrations in *Prochlorococcus*; here we assume a cytosolic concentration of 1mM for nucleotide triphosphates (e.g., ATP, GTP), probably the most abundant P-containing metabolites [S7]. Finally, all of the remaining P complement not accounted for by DNA, lipids and metabolites is assumed to be RNA, thereby closing the cellular phosphorus budget. Total RNA is assumed to be 80% rRNA, 15% tRNA and 5% mRNA [34].

Based on this cellular phosphorus budget, we calculate the amount of P in ribosomal RNA to be between 5.2 and 21.2 amol. This is sufficient to build between 598 and 2,438 ribosomes, on the basis of the size of the *Prochlorococcus* rRNA genes. At a given instant not all of those ribosomes are actively translating: some are being biosynthesized or assembled, some are awaiting transcript or tRNA binding and some are being degraded. The P budget-based calculation, however, gives us bounds for the total complement of translational capacity in a *Prochlorococcus* cell.

Comparison of transcriptome analysis by RNA sequencing and microarrays

We characterized the transcriptome dynamics of our *Prochlorococcus* culture over the diel cell cycles with two techniques: Illumina-based RNA sequencing (RNAseq) and Affymetrix microarrays. Details of analytical procedures for the two techniques are given in the Methods section. Custom Affymetrix microarrays have been used previously for transcriptomic analysis of the diel cycle in MED4 [16]. We chose to focus this paper on the results from RNAseq, because it affords a quantitative picture of the transcriptome with single-base resolution and (as performed here) strand specificity. RNAseq also does not limit the results to the regions of the genome chosen to be included in the microarray. For comparison with previous expression studies, however, we also hybridized cDNA prepared at each timepoint to our existing custom-

desighed Affymetrix arrays. The parameters of expression cycling derived from the two transcriptomic datasets are largely congruent in phase (i.e., time of peak expression) (Supporting Figure S9). RNAseq does yield consistently higher estimates than microarrays of the amplitude of mRNA abundance variation (Supporting Figure S10). Fold-change-ratio compression is often observed with microarray measurements relative to other mRNA quantification techniques, including qPCR assays [S8,S9], due to the more limited dynamic range and nonlinear signal saturation effects of fluorescence hybridization [S10]. Abundance cycles were detected for similar overall proportions of transcripts with microarrays (83%) as with RNAseq (87%), though the sensitivity and specificity of cycling detection is somewhat lower for the microarray data (Supporting Table S1). Overall, RNAseq yields a picture of the diel dynamics of the *Prochlorococcus* transcriptome that is consistent with both microarray results and previous studies [16] (particularly with regard to timing), but is more detailed, more comprehensive, and offers a wider dynamic range.

Supporting References

S1. Fu F-X, Warner ME, Zhang Y, Feng Y, Hutchins D. (2007) Effects of Increased Temperature and Co 2 on Photosynthesis, Growth, and Elemental Ratios in Marine *Synechococcus* and *Prochlorococcus* (Cyanobacteria). Journal of Phycology 43: 485–496.

S2. Heldal M, Scanlan DJ, Mann NH (2003) Elemental composition of single cells of various strains of marine *Prochlorococcus* and *Synechococcus* using X-ray microanalysis. Limnology and oceanography 48: 1732–1743.

S3. van Mooy BAS, Rocap G, Fredricks HF, Evans CT, Devol AH (2006) Sulfolipids dramatically decrease phosphorus demand by picocyanobacteria in oligotrophic marine environments. Proceedings of the National Academy of Sciences 103: 8607–8612.

S4. Ting CS, Hsieh C, Sundararaman S, Mannella C, Marko M (2007) Cryo-electron tomography reveals the comparative three-dimensional architecture of *Prochlorococcus*, a globally important marine cyanobacterium. Journal of bacteriology 189: 4485–4493.

S5. Lopez Cascales JJ, Torre JG, Marrink SJ, Berendsen HJC (1996) Molecular dynamics simulation of a charged biological membrane. Journal of Chemical Physics 104: 2713-20

S6. Nikaido, H (1996) Outer Membrane. In: Neidhart, FC, editor. *Escherichia coli* and *Salmonella typhimurium*: Cellular and Molecular Biology. Washington, D.C.: ASM Press.

S7. Bennett BD, Kimball EH, Gao M, Osterhout R, Van Dien SJ, et al. (2009) Absolute metabolite concentrations and implied enzyme active site occupancy in *Escherichia coli*. Nature chemical biology 5: 593–599.

S8. Wang, Barbacioru C, Hyland F, Xiao W, Hunkapiller KL (2006) Large scale real-time PCR validation on gene expression measurements from two commercial long-oligonucleotide microarrays. BMC Genomics 7: 59.

S9. Shi L, MAQC Consortium, et al. (2006) The MicroArray Quality Control (MAQC) project shows inter- and intraplatform reproducibility of gene expression measurements. Nature Biotechnology 24: 1151-1161.

S10. Wang Z, Gerstein M, Snyder M (2009) RNA-Seq: a revolutionary tool for transcriptomics. Nature Reviews Genetics 10: 57-63.
