# Supplementary figures and images for: Transcriptome and Proteome Dynamics of a Light-Dark Synchronized Bacterial Cell Cycle
Source: PLoS One. 2012 Aug 29;7(8):e43432. doi: 10.1371/journal.pone.0043432 (PMC3430701; doi:10.1371/journal.pone.0043432)

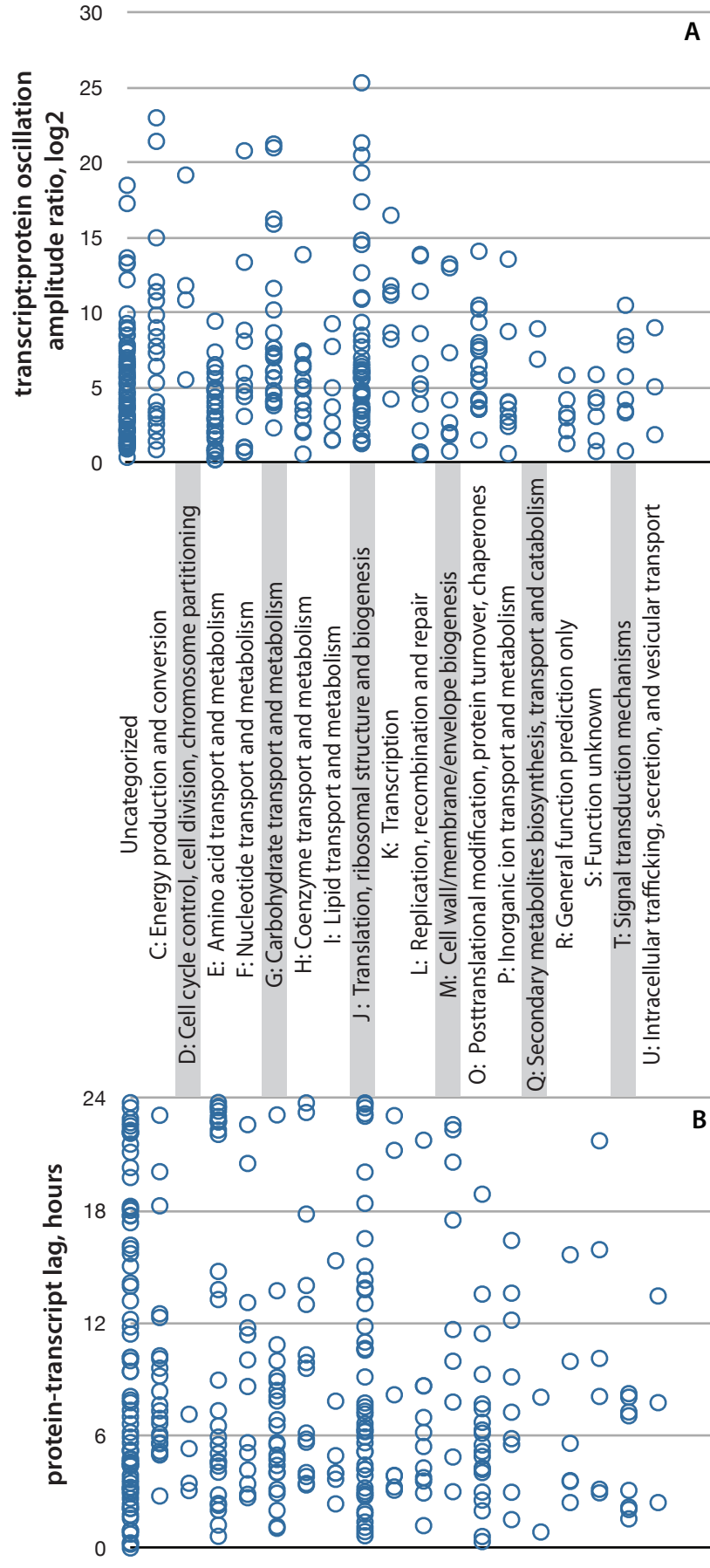

Supplement: Figure S1 — Ranges of (A) transcript∶protein oscillation amplitude ratio and (B) lag time for cycling genes grouped by Gene Ontology category. (PDF) [file pone.0043432.s002.pdf]

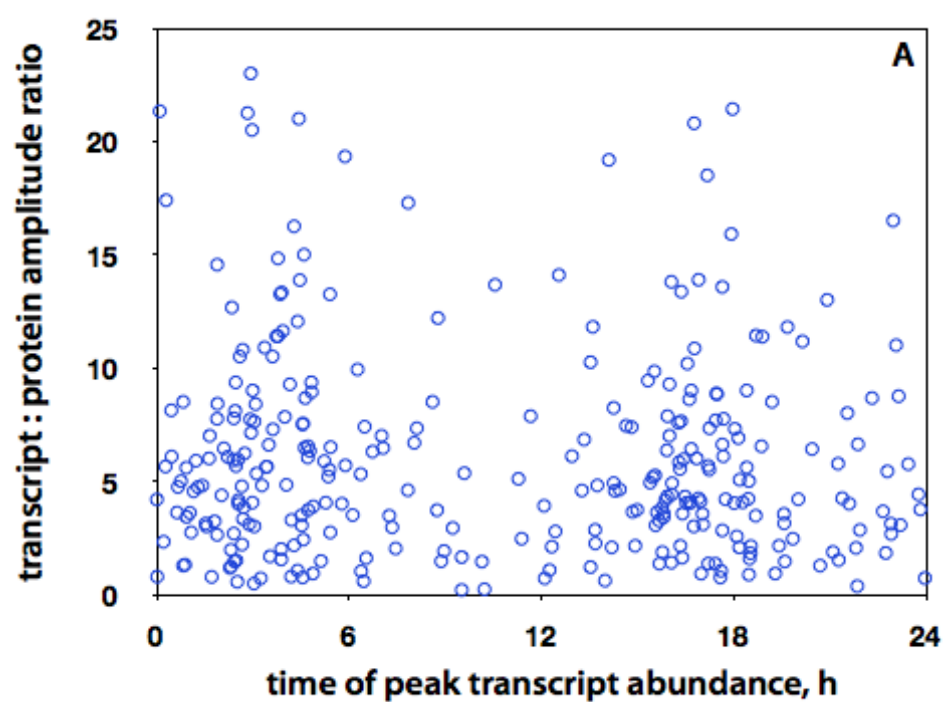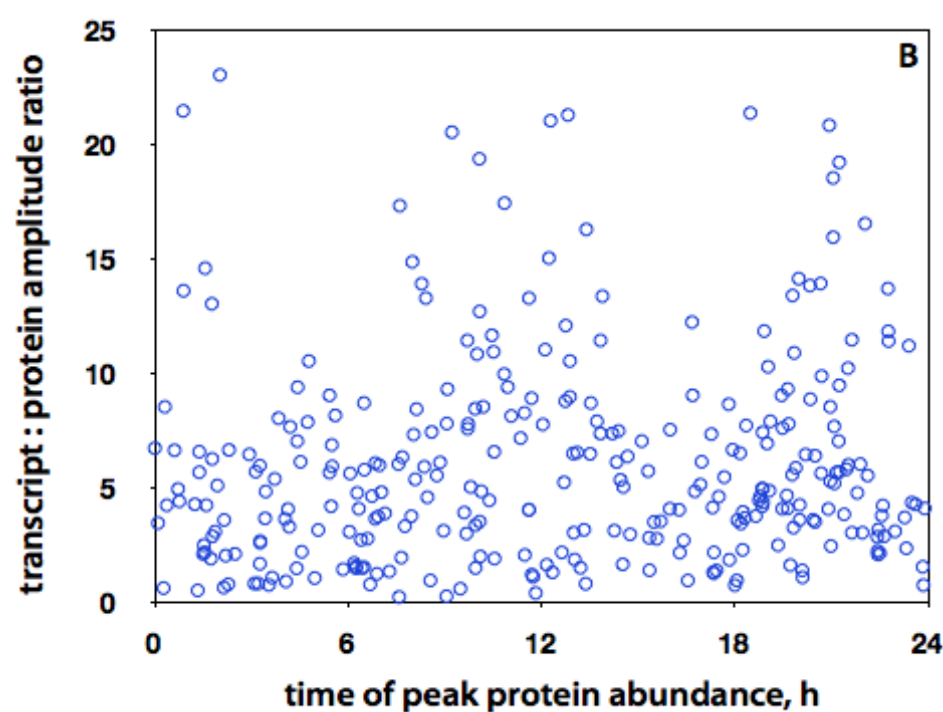

Supplement: Figure S2 — Relationships between the transcript∶protein oscillation amplitude ratio and (A) the time of peak transcript abundance and (B) the time of peak protein abundance. (PDF) [file pone.0043432.s003.pdf]

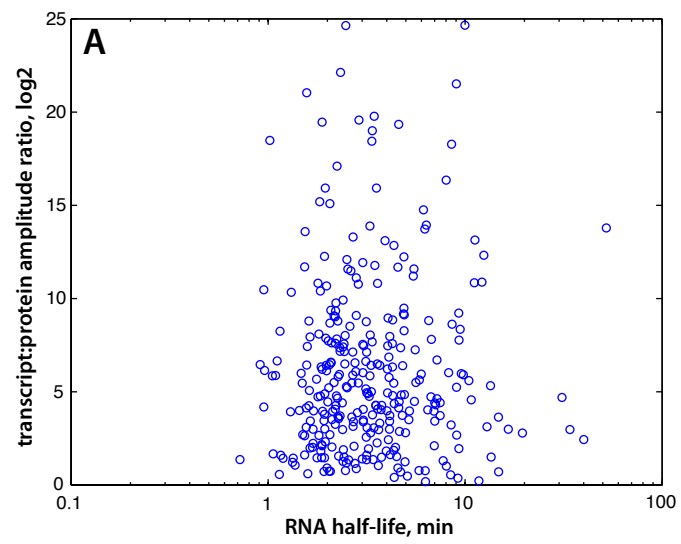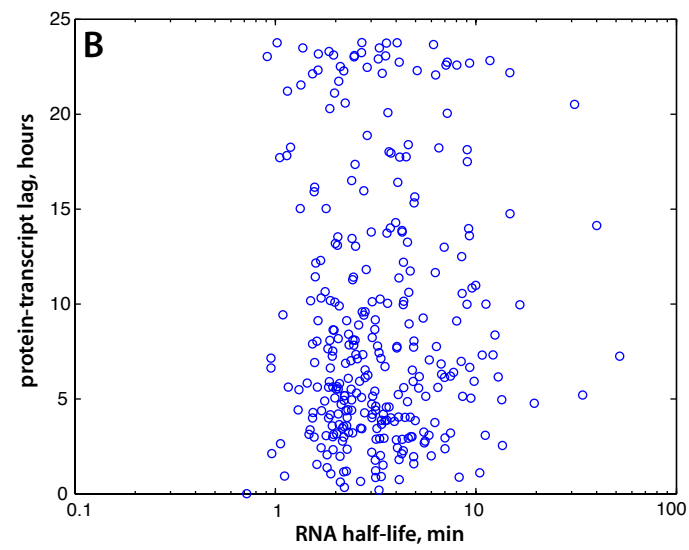

Supplement: Figure S3 — Relationships between the half-lives of transcripts in Prochlorococcus MED4 as determined by Steglich et al. [29] and (A) transcript∶protein oscillation amplitude ratio and (B) lag time between peaks of transcript and protein expression; neither cycling parameter correlates with mRNA half-life. (PDF) [file pone.0043432.s004.pdf]

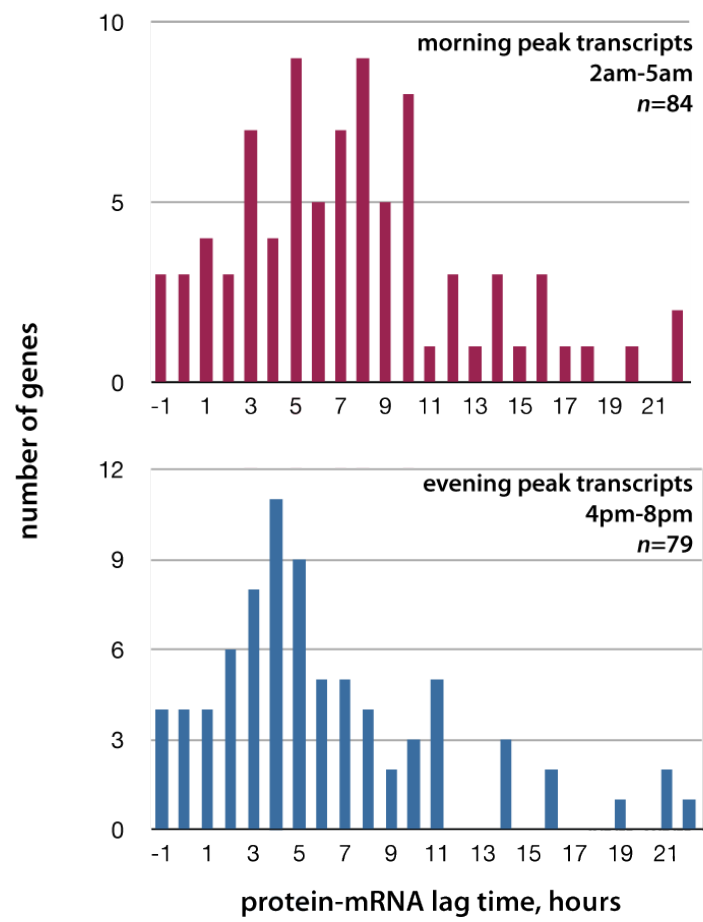

Supplement: Figure S4 — Distributions of protein-mRNA lag times for genes whose transcripts peak between 2am and 5am (upper panel), just before sunrise, and 4pm and 8pm (lower panel), around sunset. (PDF) [file pone.0043432.s005.pdf]

### Sense Coverage

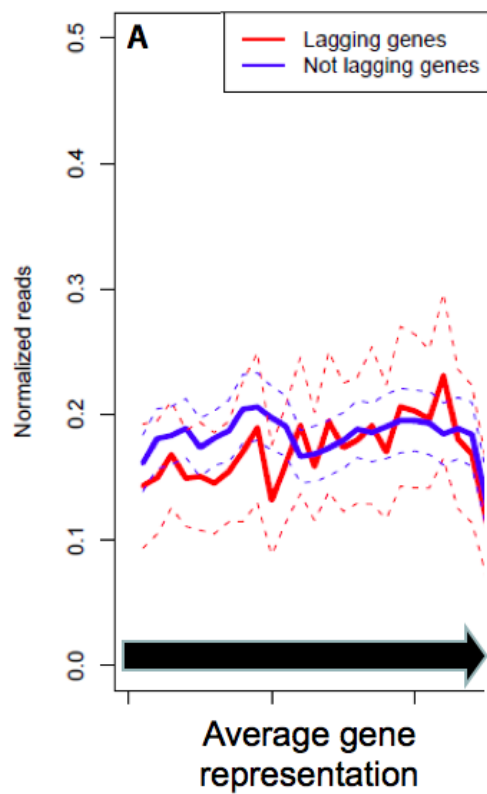

### Anti-Sense Coverage

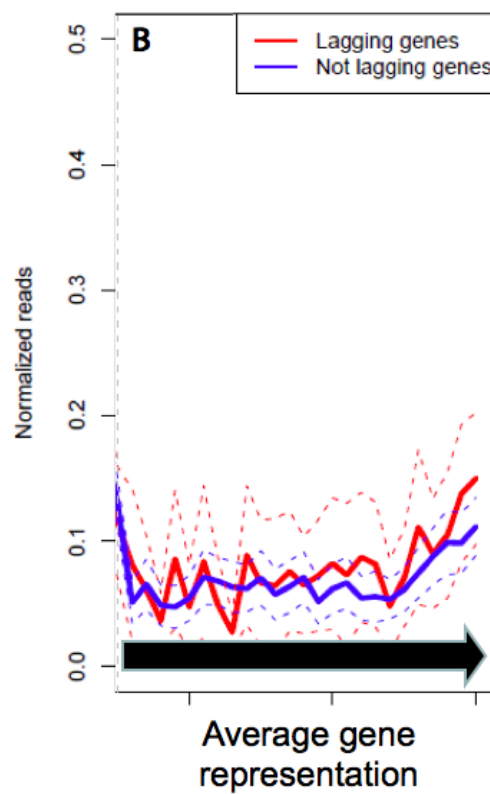

Supplement: Figure S5 — Transcriptome coverage along the length of protein-coding genes. No clear difference in depth of coverage or localization of either sense or antisense reads was observed between gene expressed in phase or not in phase at the transcript and protein levels. (PDF) [file pone.0043432.s006.pdf]

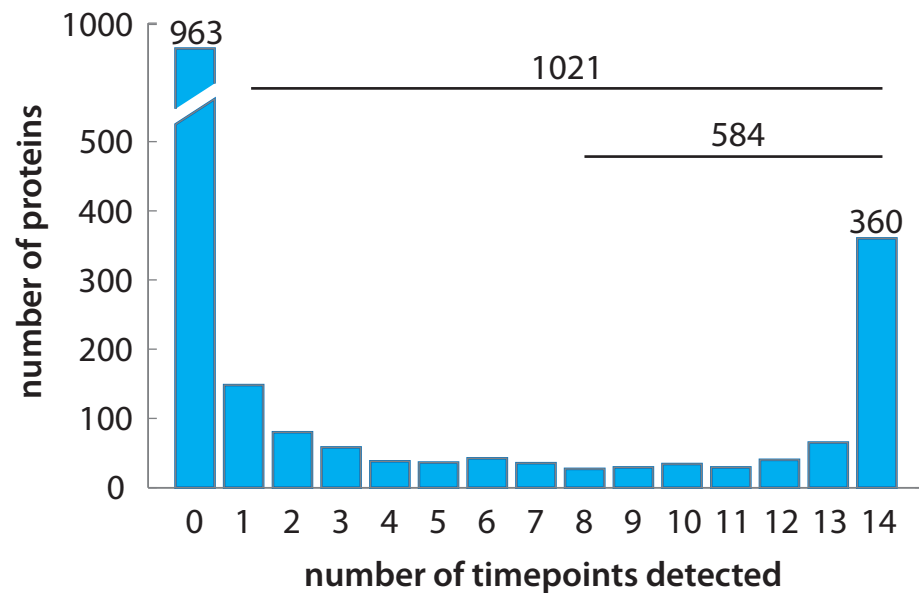

Supplement: Figure S6 — Number of timepoints at which each MED4 protein was detected. (PDF) [file pone.0043432.s007.pdf]

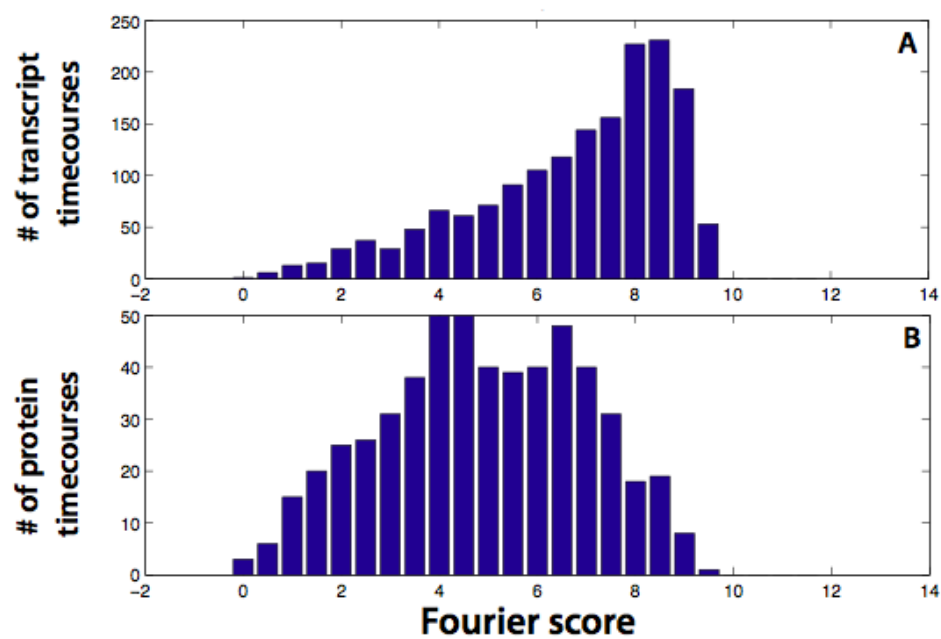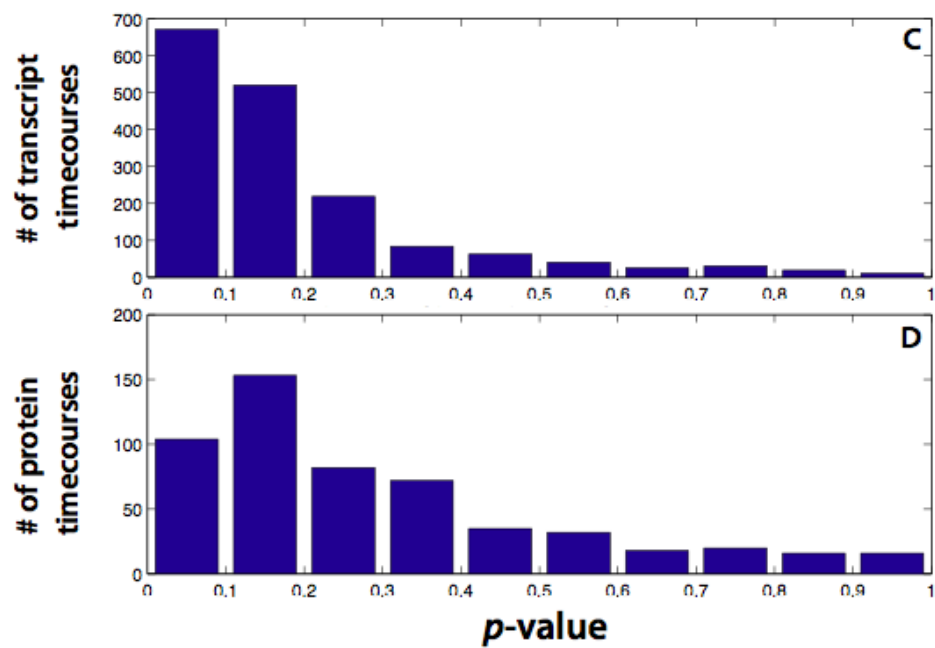

Supplement: Figure S7 — Distributions of Fourier scores (A and B) and p -values for significant diel cycling (C and D) for the 1685 transcript timecourses (A and C) and 548 protein timecourses (B and D) in the dataset. (PDF) [file pone.0043432.s008.pdf]

## Transcriptome

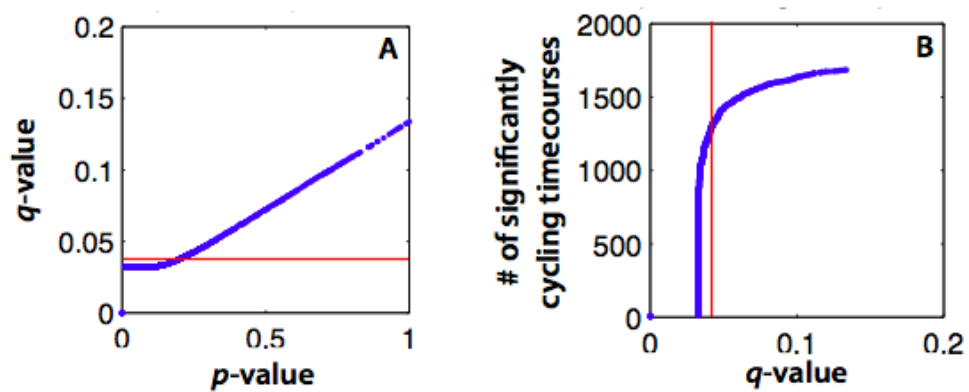

## Proteome

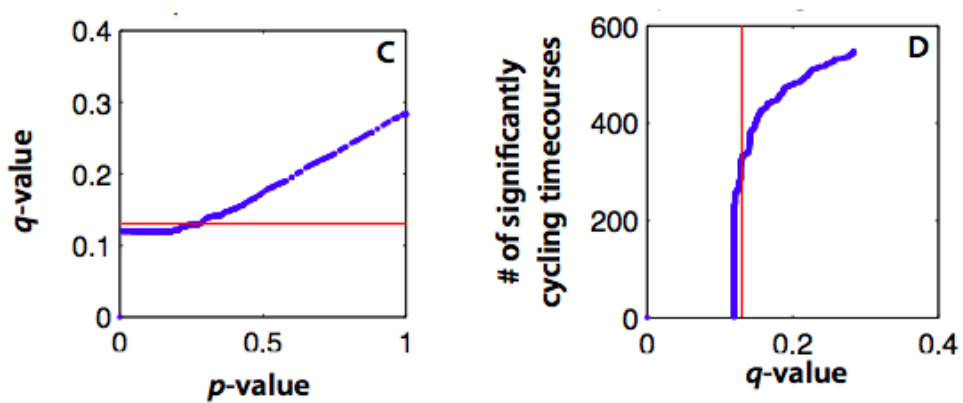

Supplement: Figure S8 — Results of QVALUE calculations (see Methods) on cycling p -value distributions for transcriptome (A and B) proteome (C and D) timecourses. Red lines indicate the chosen q-value cutoffs. (PDF) [file pone.0043432.s009.pdf]

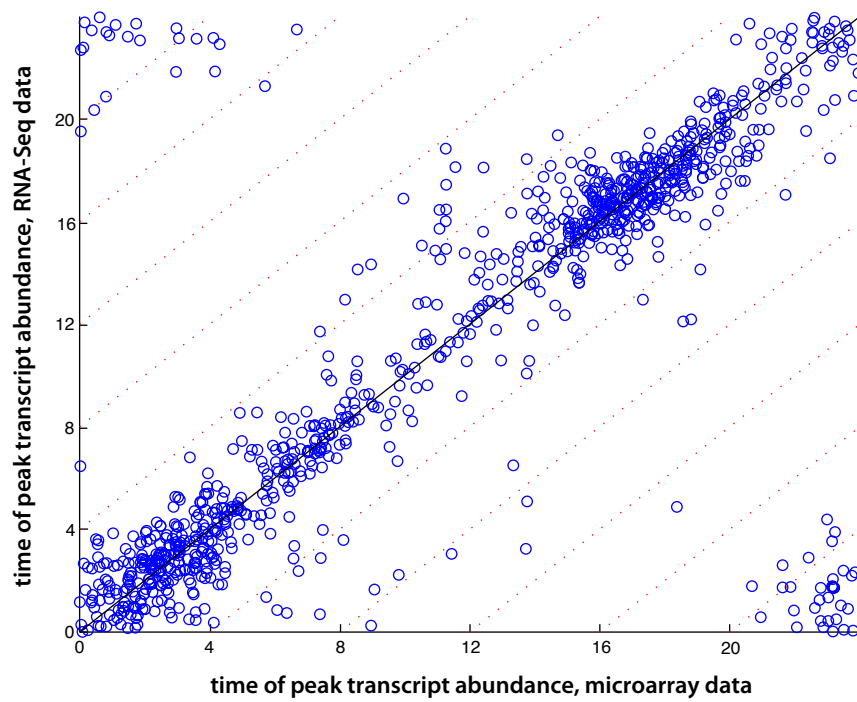

Supplement: Figure S9 — Comparison of phases (i.e., times of peak abundance) of transcripts as measured by RNA-sequencing and microarrays. (PDF) [file pone.0043432.s010.pdf]

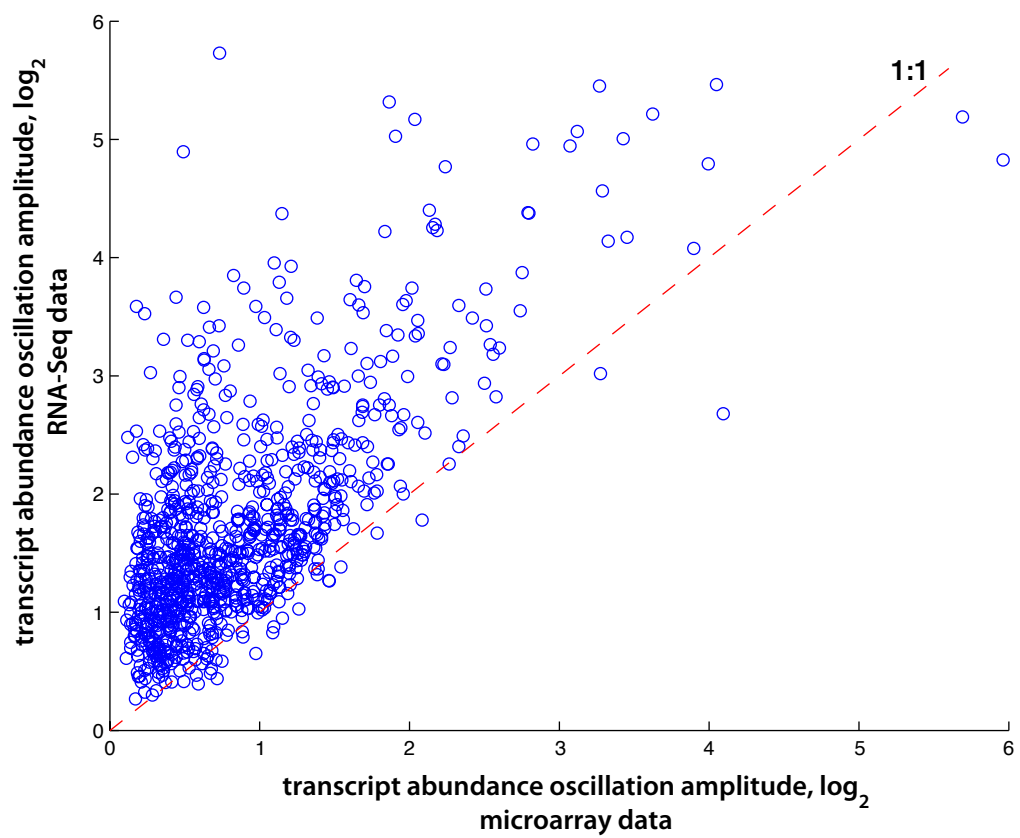

Supplement: Figure S10 — Comparison of amplitudes of transcript abundance oscillations as measured by RNA-sequencing and microarrays. (PDF) [file pone.0043432.s011.pdf]
