# Supplementary material for: Transcriptome and Proteome Dynamics of a Light-Dark Synchronized Bacterial Cell Cycle
Source: PLoS One. 2012 Aug 29;7(8):e43432. doi: 10.1371/journal.pone.0043432 (PMC3430701; doi:10.1371/journal.pone.0043432)
Supplement: Table S1 — Results of QVALUE [69] analysis of transcriptome and proteome timecourse datasets. (PDF) [file pone.0043432.s012.pdf]

	Number of Timecourses	*q*-value Cutoff	Number of Significantly Cycling Timecourses (*q*<Cutoff)	π_0	False Positives (FP)	True Negatives (TN)	True Positives (TP)	False Negatives (FN)	Fraction of Genes that are Cycling	Sensitivity	Specificity
**Transcriptome (RNAseq)**	1685	0.040	1279	0.131	4%	9%	73%	14%	87%	84%	69%
**Proteome**	548	0.130	312	0.287	13%	16%	50%	22%	71%	70%	55%
**Microarrays**	1907	0.061	1241	0.170	6%	11%	61%	22%	83%	74%	64%

Note: Based on the distributions of *q*-values, a cutoff was chosen for each dataset (Supporting Figure S4), defining the number of significantly cycling timecourses. QVALUE also calculates the parameter π_0, which estimates the total proportion of negatives (i.e., non-cycling genes) in the dataset; the fraction of cycling genes is thus 1-π_0. The proportion of false positives (FP) is equal to the *q*-value cutoff, and true negatives are given by TN= π_0-FP. True positives are given by TP=(nSignificant/nTimecourses)(1-FP), and the remainder are false negatives (FN). Two performance metrics for the detection of cycling in the two datasets are also shown: sensitivity (=TP/(TP+FN)) and specificity (=TN/(TN+FP)).
