# Supplementary material for: Transcriptome and Proteome Dynamics of a Light-Dark Synchronized Bacterial Cell Cycle
Source: PLoS One. 2012 Aug 29;7(8):e43432. doi: 10.1371/journal.pone.0043432 (PMC3430701; doi:10.1371/journal.pone.0043432)
Supplement: Table S2 — Cycling parameters of the 312 genes for which the phase and amplitude of diel oscillation could be calculated at both transcript and mRNA levels. (PDF) [file pone.0043432.s013.pdf]

Locus Tag	Gene Name	Gene Description	Time of Peak Transcript Abundance	Time of Peak Protein Abundance	Protein-Transcript Lag, hours	Transcript Amplitude, log2	Protein Amplitude, log2	Transcript: Protein Amplitude Ratio
PMED4_00001	dnaN	DNA polymerase III, beta chain (NCBI)	16.1	20.3	4.3	2.76	0.20	13.8
PMED4_00021	purL	phosphoribosylformylglycinamidine synthetase II (NCE	16.1	18.8	2.7	1.94	0.44	4.4
PMED4_00091	ftsY	signal recognition particle docking protein FtsY (NCBI)	18.2	2.0	7.8	1.62	0.32	5.1
PMED4_00121	NA	RNA-binding region RNP-1 (RNA recognition motif) (N	4	0.6	21.1	0.66	0.10	6.6
PMED4_00221	gap2	Glyceraldehyde 3-phosphate dehydrogenase(NADP+)	4.6	13.5	8.9	1.56	0.18	8.7
PMED4_00261	accB	Biotin / Lipoyl attachment:Acetyl-CoA biotin carboxyl	18.5	9.8	15.3	0.70	0.14	5.0
PMED4_00311	NA	type II secretion system protein-like (Katherine Huang)	17.8	0.4	6.6	2.94	0.70	4.2
PMED4_00341	DHSS	soluble hydrogenase small subunit (NCBI)	15.9	21	4.6	3.18	0.50	6.4
PMED4_00361	guaA	Glutamine amidotransferase class-I:GMP synthase (N	17.5	20.4	2.9	2.12	0.24	8.8
PMED4_00441	alaS	Alanyl-tRNA synthetase (NCBI)	21.9	2	3.9	1.08	0.38	2.8
PMED4_00461	ndk	Nucleoside diphosphate kinase (NCBI)	2.5	11.1	8.6	1.62	0.20	8.1
PMED4_00481	gatB	Glutamyl-tRNA (Gln) amidotransferase subunit B (NCE	15.6	3.4	11.8	0.58	0.16	3.6
PMED4_00551	NA	conserved hypothetical protein (NCBI)	16.3	12.6	20.3	2.14	1.00	2.1
PMED4_00601	NA	Uncharacterized conserved protein (Katherine Huang)	15.9	18.9	3.0	2.16	0.50	4.3
PMED4_00621	accC	acetyl-CoA carboxylase, biotin carboxylase subunit (N	2.4	6.4	4.0	0.86	0.32	2.7
PMED4_00651	NA	conserved hypothetical protein (NCBI)	17.2	15.4	22.2	1.28	0.94	1.4
PMED4_00771	NA	Uncharacterized protein conserved in bacteria (Kather	17.1	9.0	15.9	5.06	1.64	3.1
PMED4_00791	pgm	Phosphoglucomutase (NCBI)	13.0	17.0	4.0	1.22	0.20	6.1
PMED4_01021	NA	possible RND family outer membrane efflux protein (N	1.7	13.4	11.7	1.14	1.48	0.8
PMED4_01211	crtQ	zeta-carotene desaturase (NCBI)	4.6	2.3	21.7	0.88	1.18	0.7
PMED4_01301	NA	conserved hypothetical protein in cyanobacteria (NCB	2.3	17.4	15.0	1.38	1.12	1.2
PMED4_01341	rpaA	two-component response regulator (NCBI)	11.7	13.7	2.1	2.36	0.30	7.9
PMED4_01401	rpaB	two-component response regulator (NCBI)	2	10.0	8.1	2.02	0.24	8.4
PMED4_01481	NA	RNA-binding region RNP-1 (RNA recognition motif) (N	3.5	6.2	2.7	1.14	0.68	1.7
PMED4_01561	ndhD	putative NADH dehydrogenase subunit (chain 4) (NCB	16.7	2.9	10.3	3.34	0.52	6.4
PMED4_01651	ndhI	putative NADH Dehydrogenase subunit (NCBI)	18.5	4.1	9.6	1.08	1.24	0.9
PMED4_01671	gltA	Citrate synthase (NCBI)	18.0	0.9	6.9	3.00	0.14	21.4
PMED4_01721	cysC	Adenylylsulfate kinase (NCBI)	1.1	6.6	5.5	1.04	0.38	2.7
PMED4_01751	NA	two-component response regulator (NCBI)	18.5	20.0	1.6	1.18	0.28	4.2
PMED4_01941	nadC	Nicotinate-nucleotide pyrophosphorylase:Quinolinate	6.5	0.3	17.8	0.50	0.86	0.6
PMED4_01951	thdF	putative thiophen / furan oxidation protein (NCBI)	3.1	13.0	10.0	1.80	0.60	3.0
PMED4_02011	pgk	Phosphoglycerate kinase (NCBI)	7.0	15.1	8.1	2.94	0.42	7.0
PMED4_02081	rplJ	50S ribosomal protein L10 (NCBI)	1.9	1.6	23.7	2.62	0.18	14.6
PMED4_02101	rplK	50S ribosomal protein L11 (NCBI)	23.1	12.1	13.1	1.10	0.10	11.0
PMED4_02111	nusG	transcription antitermination protein, NusG (NCBI)	22.3	7	8.2	1.04	0.12	8.7

Locus Tag	Gene Name	Gene Description	Time of Peak Transcript Abundance	Time of Peak Protein Abundance	Protein-Transcript Lag, hours	Transcript Amplitude, log2	Protein Amplitude, log2	Transcript: Protein Amplitude Ratio
PMED4_02141	eno	Enolase (NCBI)	17.9	21.1	3.1	3.50	0.22	15.9
PMED4_02171	trxB	FAD-dependent pyridine nucleotide-disulphide oxidor	13.5	19.0	5.5	2.46	0.24	10.3
PMED4_02201	NA	putative sulfate transporter (NCBI)	4.5	6.1	1.5	1.40	0.46	3.0
PMED4_02281	psbA	Photosystem II PsbA protein (D1) (NCBI)	11.4	1.5	14.1	2.70	1.10	2.5
PMED4_02291	aroC	Chorismate synthase (NCBI)	19	8.6	13.3	1.56	1.70	0.9
PMED4_02311	ftsH2	cell division protein FtsH2 (NCBI)	5.4	13.1	7.7	1.04	0.16	6.5
PMED4_02321	cysD	ATP-sulfurylase (NCBI)	4.6	21.0	16.4	1.16	0.48	2.4
PMED4_02331	psbO	Photosystem II manganese-stabilizing protein (NCBI)	6.4	14.5	8.1	1.38	0.26	5.3
PMED4_02431	ileS	Isoleucyl-tRNA synthetase (NCBI)	21.9	17.9	20.1	1.06	0.16	6.6
PMED4_02481	thy1	possible Thy1 protein homolog (NCBI)	16.4	20	3.4	2.94	0.22	13.4
PMED4_02491	dcd	dCTP Deaminase (NCBI)	16.4	21.5	5.1	3.46	0.58	6.0
PMED4_02631	glyA	Serine hydroxymethyltransferase (SHMT) (NCBI)	14.3	18.9	4.6	2.26	0.46	4.9
PMED4_02681	amt1	Ammonium transporter family (NCBI)	17.7	1	7.3	3.80	0.28	13.6
PMED4_02781	NA	probable oxidoreductase (NCBI)	4.2	7.8	3.6	1.84	0.56	3.3
PMED4_02861	hisB	Imidazoleglycerol-phosphate dehydratase (NCBI)	23.2	23.0	23.8	1.04	0.34	3.1
PMED4_02871	fabI	enoyl-[acyl-carrier-protein] reductase (NCBI)	1.9	9.7	7.8	1.24	0.16	7.8
PMED4_02941	NA	possible ABC transporter (NCBI)	18.2	19.0	0.9	1.38	0.20	6.9
PMED4_03021	psbE	Cytochrome b559 alpha-subunit (NCBI)	10.6	22.8	12.2	1.64	0.12	13.7
PMED4_03041	psbL	photosystem II PsbL protein (NCBI)	8.0	0.0	16.0	1.74	0.26	6.7
PMED4_03061	NA	5'-methylthioadenosine phosphorylase (NCBI)	17.6	3.7	10.0	1.08	1.04	1.0
PMED4_03171	metK	S-adenosylmethionine synthetase (NCBI)	5.4	8.8	3.4	0.88	0.16	5.5
PMED4_03181	rps1a, rpsA	30S ribosomal protein S1, homolog A (NCBI)	2.5	7.0	4.4	2.26	0.38	5.9
PMED4_03271	minE	possible septum site-determining protein MinE (NCBI)	14.1	21.3	7.1	4.22	0.22	19.2
PMED4_03281	minD	putative septum site-determining protein MinD (NCBI)	13.6	18.9	5.3	4.96	0.42	11.8
PMED4_03321	petB	Cytochrome b6 (NCBI)	13.5	7	17.4	1.54	1.28	1.2
PMED4_03341	NA	putative neutral invertase-like protein (NCBI)	19.6	18.1	22.5	1.42	0.40	3.6
PMED4_03361	psaE	Photosystem I PsaE protein (subunit IV) (NCBI)	6.7	7.7	1.0	1.26	0.20	6.3
PMED4_03371	NA	possible LysM domain (NCBI)	16	14.5	22.1	3.14	1.94	1.6
PMED4_03501	NA	conserved hypothetical (NCBI)	4	20.1	15.7	0.42	0.40	1.1
PMED4_03911	NA	possible Malic enzyme (NCBI)	17.2	21.1	3.9	5.18	0.28	18.5
PMED4_03941	NA	conserved hypothetical protein (NCBI)	18.0	4.1	10.1	2.66	0.66	4.0
PMED4_04231	spnII-interr	possible uncharacterized restriction enzyme, interrupte	17.7	22.4	4.8	2.54	0.90	2.8
PMED4_04411	lrtA	light repressed protein A homolog (NCBI)	16.1	5.9	13.8	1.82	1.30	1.4
PMED4_04441	NA	conserved hypothetical protein (NCBI)	15.9	19.1	3.2	2.36	0.30	7.9
PMED4_04501	cysK1	O-acetylserine (thiol)-lyase A (NCBI)	18.9	1.4	6.5	1.70	0.26	6.5

Locus Tag	Gene Name	Gene Description	Time of Peak Transcript Abundance	Time of Peak Protein Abundance	Protein-Transcript Lag, hours	Transcript Amplitude, log2	Protein Amplitude, log2	Transcript: Protein Amplitude Ratio
PMED4_04531	rpsD	30S ribosomal protein S4 (NCBI)	2.5	3.1	0.7	1.36	0.24	5.7
PMED4_04601	NA	Conserved hypothetical protein (Katherine Huang)	16.8	22.0	5.2	1.38	0.46	3.0
PMED4_04831	topA	Prokaryotic DNA topoisomerase (NCBI)	17.7	2.3	8.7	1.72	0.26	6.6
PMED4_05011	fabG	3-oxoacyl-[acyl-carrier protein] reductase (NCBI)	4.2	9.1	4.9	2.04	0.22	9.3
PMED4_05051	ppx	putative exopolyphosphatase (NCBI)	6.4	5.0	22.6	1.12	1.10	1.0
PMED4_05061	NA	conserved hypothetical protein (NCBI)	18.4	17	22.3	2.52	0.28	9.0
PMED4_05101	petC	Rieske iron-sulfur protein (NCBI)	10.2	4.4	18.3	1.04	0.72	1.4
PMED4_05171	psaF	Photosystem I PsaF protein (subunit III) (NCBI)	7.8	6.8	22.9	0.92	0.20	4.6
PMED4_05211	gltX	Glutamyl-tRNA synthetase (NCBI)	22.8	13.0	14.3	0.84	0.46	1.8
PMED4_05231	rplS	Ribosomal protein L19 (NCBI)	2.7	7.2	4.4	1.46	0.38	3.8
PMED4_05281	NA	conserved hypothetical protein (NCBI)	16.3	19.5	3.2	2.12	0.28	7.6
PMED4_05311	hflC	Band 7 protein (NCBI)	12.6	20.0	7.4	2.82	0.20	14.1
PMED4_05421	cxp	Carboxypeptidase Taq (M32) metallopeptidase (NCBI)	9.5	7.6	22.1	0.18	0.94	0.2
PMED4_05451	NA	Putative principal RNA polymerase sigma factor (NCB	21.4	1.3	3.9	2.46	0.58	4.2
PMED4_05551	purA	Adenylosuccinate synthetase (NCBI)	2.9	14.3	11.4	0.68	0.22	3.1
PMED4_05561	psb27	possible Photosystem II reaction center Psb27 protein	4.9	9.6	4.7	1.32	0.34	3.9
PMED4_05571	proS	Prolyl-tRNA synthetase (NCBI)	21.3	21.5	0.2	1.50	0.26	5.8
PMED4_05641	gpmB	possible alpha-ribazole-5'-P phosphatase (NCBI)	2.6	5.5	2.9	1.08	0.26	4.2
PMED4_05681	tal	Transaldolase (NCBI)	15.8	21	5.6	3.88	1.02	3.8
PMED4_05701	frr	Ribosome recycling factor (NCBI)	18.7	21	1.8	1.18	0.34	3.5
PMED4_05751	ilvH	Acetolactate synthase large subunit (NCBI)	16.9	22	5.1	1.56	0.26	6.0
PMED4_05791	rps1b, rps/	30S ribosomal protein S1 homolog B, putative Nbp1 (I	1.8	19.8	18.0	0.58	0.18	3.2
PMED4_05811	NA	conserved hypothetical protein (NCBI)	12.3	11.8	23.5	0.62	0.58	1.1
PMED4_05921	chlL	Protochlorophyllide reductase iron-sulfur ATP-binding	14.0	2.2	12.2	0.74	1.22	0.6
PMED4_05931	chlB	Light-independent protochlorophyllide reductase subu	18.3	24	5.6	2.60	0.64	4.1
PMED4_05941	chlN	Light-independent protochlorophyllide reductase subu	18	3.3	9.2	2.66	1.04	2.6
PMED4_05981	ccmK	carboxysome shell protein CsoS1 (NCBI)	4.8	12.9	8.1	2.68	0.30	8.9
PMED4_05991	rbcL	Ribulose bisphosphate carboxylase, large chain (NCB	4.3	13	9.1	5.20	0.32	16.3
PMED4_06001	rbcS	Ribulose bisphosphate carboxylase, small chain (NCB	4	12.8	8.4	4.82	0.40	12.1
PMED4_06011	csoS2	carboxysome shell protein CsoS2 (NCBI)	4	14	10.0	4.80	0.36	13.3
PMED4_06091	hisG	possible ATP phosphoribosyltransferase (NCBI)	2.7	7.0	4.4	1.24	0.26	4.8
PMED4_06251	NA	conserved hypothetical protein (NCBI)	14.9	18.3	3.5	5.32	1.46	3.6
PMED4_06261	NA	Putative type II alternative sigma factor, sigma70 famil	14.3	11.5	21.2	4.94	0.60	8.2
PMED4_06321	hemE	Uroporphyrinogen decarboxylase (URO-D) (NCBI)	4.7	10.5	5.8	1.96	0.30	6.5
PMED4_06331	glgB	1,4-alpha-glucan branching enzyme (NCBI)	3.0	11.4	8.4	2.14	0.30	7.1

Locus Tag	Gene Name	Gene Description	Time of Peak Transcript Abundance	Time of Peak Protein Abundance	Protein-Transcript Lag, hours	Transcript Amplitude, log2	Protein Amplitude, log2	Transcript: Protein Amplitude Ratio
PMED4_06391	proA	Gamma-glutamyl phosphate reductase (NCBI)	4	3.2	23.0	0.58	0.74	0.8
PMED4_06471	trpS	Tryptophanyl-tRNA synthetase (NCBI)	0.5	14.3	13.9	1.46	0.24	6.1
PMED4_06671	sds	polyprenyl synthetase; solanesyl diphosphate synthas	6.1	15.7	9.6	0.98	0.28	3.5
PMED4_06681	acs	acetyl-coenzyme A synthetase (NCBI)	15.0	18.7	3.7	0.82	0.22	3.7
PMED4_06911	NA	conserved hypothetical protein (NCBI)	2.7	22.4	19.8	0.66	0.30	2.2
PMED4_07151	NA	Conserved hypothetical protein (Katherine Huang)	7.4	9.7	2.3	1.48	0.50	3.0
PMED4_07171	nrdJ	ribonucleotide reductase (Class II) (NCBI)	15.7	20	4.4	4.18	3.08	1.4
PMED4_07381	aroB	3-dehydroquinate synthase (NCBI)	19.9	19.4	23.5	0.54	0.22	2.5
PMED4_07841	NA	ABC transporter, substrate binding protein, phosphate	23.2	12.8	13.6	0.70	0.08	8.8
PMED4_07981	NA	hypothetical (NCBI)	3.4	21.2	17.7	1.24	0.22	5.6
PMED4_08091	NA	possible Major surface glycoprotein (NCBI)	7.9	7.6	23.8	2.42	0.14	17.3
PMED4_08181	NA	possible Alpha-2-macroglobulin family N-termin (NCB	13.3	6	16.2	0.82	0.12	6.8
PMED4_08261	NA	conserved hypothetical protein (NCBI)	2	13.5	11.4	1.16	0.18	6.4
PMED4_08271	NA	Clp protease subunit (NCBI)	17.0	17.3	0.4	0.82	0.20	4.1
PMED4_08281	NA	FtsH ATP-dependent protease homolog (NCBI)	4.6	9.7	5.2	1.66	0.22	7.5
PMED4_08381	rpsB	30S ribosomal protein S2 (NCBI)	3.8	8	4.2	1.78	0.12	14.8
PMED4_08431	sir	Ferredoxin-sulfite reductase (NCBI)	16.7	19.7	3.0	1.30	0.32	4.1
PMED4_08451	chlP	Aromatic-ring hydroxylase (flavoprotein monooxygena	14	3	12.3	1.12	0.54	2.1
PMED4_08471	typA	tyrosine binding protein (NCBI)	3	4.8	2.2	0.84	0.08	10.5
PMED4_08511	rpe	Ribulose-phosphate 3-epimerase (NCBI)	3.1	4.2	1.2	2.44	0.32	7.6
PMED4_08521	glpX	Fructose-1,6-bisphosphatase/sedoheptulose-1, 7-bis	4.4	12.3	7.9	2.10	0.10	21.0
PMED4_08531	hemA	glutamyl-tRNA reductase (NCBI)	3.0	16.0	13.0	1.70	0.42	4.0
PMED4_08541	glgC	ADP-glucose pyrophosphorylase (NCBI)	3.9	10.5	6.5	1.86	0.16	11.6
PMED4_08551	gnd	6-phosphogluconate dehydrogenase (NCBI)	16.6	21.5	5.0	3.26	0.32	10.2
PMED4_08621	NA	conserved hypothetical protein (NCBI)	6.5	12.2	5.6	0.80	0.50	1.6
PMED4_08661	NA	phosphoribosylformylglycinamidine synthase (NCBI)	16.8	20.9	4.2	2.08	0.10	20.8
PMED4_08671	cbbA	Fructose-bisphosphate/sedoheptulose-1, 7-bisphospl	3.3	10.1	6.9	2.50	0.52	4.8
PMED4_08711	prkB	phosphoribulokinase (NCBI)	5	12.2	7.6	2.10	0.14	15.0
PMED4_08831	NA	possible mRNA binding protein (NCBI)	2.3	10.1	7.8	1.10	0.56	2.0
PMED4_08901	NA	ferritin (NCBI)	5.8	11.6	5.8	0.72	0.18	4.0
PMED4_09191	carB	carbamoyl-phosphate synthase, large subunit (NCBI)	16.6	19.5	2.8	1.62	0.40	4.1
PMED4_09231	tpi	Triosephosphate isomerase (NCBI)	5.3	6.3	1.1	1.78	0.44	4.0
PMED4_09251	chlH	protoporphyrin IX magnesium chelatase, subunit chlH	7.5	11.5	4.0	1.50	0.74	2.0
PMED4_09341	mfd	Transcriptional-repair coupling factor (NCBI)	2.5	9.5	7.0	0.72	1.30	0.6
PMED4_09391	PNIL34,AT	phytochrome-regulated gene (NCBI)	8.9	6.4	21.6	1.46	1.00	1.5

Locus Tag	Gene Name	Gene Description	Time of Peak Transcript Abundance	Time of Peak Protein Abundance	Protein-Transcript Lag, hours	Transcript Amplitude, log2	Protein Amplitude, log2	Transcript: Protein Amplitude Ratio
PMED4_09741	NA	possible Fusion glycoprotein F0. (NCBI)	4.9	18.1	13.2	1.12	1.22	0.9
PMED4_09771	metG	Methionyl-tRNA synthetase (NCBI)	5.9	10.1	4.2	1.16	0.06	19.3
PMED4_09811	pheT	Phenylalanyl-tRNA synthetase beta chain (NCBI)	0	18.5	18.4	1.28	0.06	21.3
PMED4_09881	metH	putative methionine synthase (NCBI)	10.2	9.1	22.8	0.30	1.34	0.2
PMED4_09891	ilvE	putative Branched-chain amino acid aminotransferase	19	19.7	1.2	0.38	0.24	1.6
PMED4_09941	NA	Conserved protein/domain typically associated with fla	14.9	17.4	2.4	0.60	0.28	2.1
PMED4_09971	NA	conserved hypothetical protein (NCBI)	21.9	11.8	14.0	0.26	0.72	0.4
PMED4_10001	leuS	Leucyl-tRNA synthetase (NCBI)	14	17.5	3.2	0.64	0.14	4.6
PMED4_10011	pgi	Phosphoglucose isomerase (PGI) (NCBI)	13.3	18.8	5.5	1.10	0.24	4.6
PMED4_10031	argC	N-acetyl-gamma-glutamyl-phosphate reductase (Kath	18.5	17.8	23.3	2.00	1.10	1.8
PMED4_10051	NA	Cyclophilin-type peptidyl-prolyl cis-trans isomerase (N	4.6	16	11.4	1.50	0.20	7.5
PMED4_10081	dnaK	Molecular chaperone DnaK, heat shock protein hsp70	16.9	23.6	6.7	1.44	0.34	4.2
PMED4_10131	rpmB	50S ribosomal protein L28 (NCBI)	4.1	19.1	15.0	1.16	0.24	4.8
PMED4_10171	psaK	Photosystem I PsaK protein (subunit X) (NCBI)	8.6	10	1.6	1.36	0.16	8.5
PMED4_10181	Dxs	1-deoxy-D-xylulose 5-phosphate synthase (NCBI)	4.5	8.3	3.8	2.22	0.16	13.9
PMED4_10231	pykF	Pyruvate kinase (NCBI)	3.6	8.0	4.4	1.02	0.14	7.3
PMED4_10311	glnA	Glutamine synthetase, glutamate--ammonia ligase (NC	14.8	18.9	4.1	3.10	0.42	7.4
PMED4_10371	psb28	possible Photosystem II reaction center Psb28 protein	5.9	15.3	9.4	1.48	0.26	5.7
PMED4_10411	pdhB	Pyruvate dehydrogenase E1 beta subunit (NCBI)	2.9	2.0	23.1	1.38	0.06	23.0
PMED4_10521	NA	possible cAMP phosphodiesterases class-II (NCBI)	14.5	19.6	5.2	1.48	0.32	4.6
PMED4_10541	rpsO	30S Ribosomal protein S15 (NCBI)	3.4	10.5	7.2	2.18	0.20	10.9
PMED4_10571	gatA	Glutamyl-tRNA(Gln) amidotransferase A subunit (NCB	19.6	22.5	2.9	1.38	0.44	3.1
PMED4_10621	NA	putative Anthranilate synthase component II (NCBI)	15.3	21.3	5.9	3.02	0.32	9.4
PMED4_10751	ureC	Urease alpha subunit (NCBI)	18.4	20.7	2.3	2.24	0.40	5.6
PMED4_11071	rpsU	30S Ribosomal protein S21 (NCBI)	4.7	6.9	2.1	2.90	0.48	6.0
PMED4_11181	NA	conserved hypothetical protein (NCBI)	17.4	18.4	0.9	3.38	0.44	7.7
PMED4_11211	NA	possible Protein of unknown function DUF67 (NCBI)	6.3	10.9	4.6	2.78	0.28	9.9
PMED4_11321	purT	GAR transformylase 2 (NCBI)	12.1	23.9	11.8	0.34	0.48	0.7
PMED4_11341	btuE	Glutathione peroxidase (NCBI)	4.6	18.2	13.6	2.46	0.38	6.5
PMED4_11351	NA	Conserved hypothetical membrane protein (Katherine	1.5	13.3	11.8	0.50	0.16	3.1
PMED4_11871	NA	conserved hpothetical protein (NCBI)	5.2	8.4	3.1	2.00	0.34	5.9
PMED4_12011	chlI	Protoporphyrin IX Magnesium chelatase, ChlI subunit	4.8	14.7	9.9	1.52	0.24	6.3
PMED4_12071	trxA	Thioredoxin (NCBI)	12.4	15.4	2.9	0.50	0.18	2.8
PMED4_12081	guaB	putative IMP dehydrogenase (NCBI)	16	20	3.6	1.40	0.24	5.8
PMED4_12091	gyrA	DNA gyrase/topoisomerase IV, subunit A (NCBI)	19	21.6	2.9	1.60	0.14	11.4

Locus Tag	Gene Name	Gene Description	Time of Peak Transcript Abundance	Time of Peak Protein Abundance	Protein-Transcript Lag, hours	Transcript Amplitude, log2	Protein Amplitude, log2	Transcript: Protein Amplitude Ratio
PMED4_12161	ispA	Polyprenyl synthetase (NCBI)	21.6	11.6	14.0	0.56	0.14	4.0
PMED4_12191	NA	Putative glucose 6-phosphate dehydrogenase effecto	17.7	4.5	10.9	2.56	0.42	6.1
PMED4_12201	zwf	Glucose-6-phosphate dehydrogenase (NCBI)	17.2	21.3	4.1	2.38	0.42	5.7
PMED4_12251	NA	possible Villin headpiece domain (NCBI)	4.3	4.6	0.3	0.52	0.24	2.2
PMED4_12261	prsA	Ribose-phosphate pyrophosphokinase (NCBI)	15.5	21	5.6	1.96	0.38	5.2
PMED4_12301	cad	Orn/Lys/Arg decarboxylases family 1 (NCBI)	3.0	1.3	22.3	0.48	1.00	0.5
PMED4_12441	psbP	photosystem II oxygen evolving complex protein PsbF	4.7	8.0	3.2	1.34	0.36	3.7
PMED4_12471	srmB	putative ATP-dependent RNA helicase (NCBI)	24.0	3.6	3.6	1.46	2.04	0.7
PMED4_12531	pdxJ	Pyridoxal phosphate biosynthetic protein PdxJ (NCBI)	15.6	1.9	10.3	1.22	0.40	3.1
PMED4_12571	NA	Glutaredoxin-related protein (NCBI)	23	17.7	18.9	0.76	0.14	5.4
PMED4_12591	NA	two-component response regulator (NCBI)	7.3	16	8.2	1.18	0.34	3.5
PMED4_13031	pntB	putative nicotinamide nucleotide transhydrogenase, su	15.7	4.2	12.5	2.54	0.78	3.3
PMED4_13051	pntA	putative nicotinamide nucleotide transhydrogenase, su	15.5	21	5.2	3.74	0.38	9.8
PMED4_13161	NA	photosystem I assembly related protein Ycf4 (NCBI)	5.4	15.6	10.2	0.82	0.30	2.7
PMED4_13171	psbD	Photosystem II PsbD protein (D2) (NCBI)	10	3.7	18.1	2.14	0.40	5.4
PMED4_13181	psbC	Photosystem II PsbC protein (CP43) (NCBI)	8.8	6.9	22.2	1.56	0.42	3.7
PMED4_13291	glyQ	Glycyl-tRNA synthetase alpha subunit (NCBI)	20	6.3	10.7	1.16	0.80	1.5
PMED4_13301	NA	conserved hypothetical protein (NCBI)	17.5	12	18.2	2.84	0.32	8.9
PMED4_13311	NA	Macrophage migration inhibitory factor family (NCBI)	16.5	20.9	4.4	2.18	0.54	4.0
PMED4_13361	isiB	Flavodoxin (NCBI)	8.1	14.2	6.1	1.32	0.18	7.3
PMED4_13451	sppA	signal peptide peptidase SppA (protease IV) (NCBI)	17.2	22.1	4.9	1.54	0.28	5.5
PMED4_13501	NA	conserved hypothetical protein (NCBI)	2.3	11.7	9.4	1.10	0.94	1.2
PMED4_13521	NA	ATPase of the AAA+ family (Katherine Huang)	21.3	23.8	2.6	1.30	0.86	1.5
PMED4_13551	rpsN	30S Ribosomal protein S14 (NCBI)	2.8	1.8	23.0	1.12	0.18	6.2
PMED4_13561	pnp	polyribonucleotide nucleotidyltransferase (NCBI)	3.0	9.2	6.3	1.64	0.08	20.5
PMED4_13651	NA	Glycosyl transferase, family 2 (NCBI)	3.9	2.2	22.3	1.92	0.96	2.0
PMED4_13771	NA	conserved hypothetical protein (NCBI)	5.1	13.2	8.1	1.00	0.68	1.5
PMED4_13781	NA	Glycosyl transferases group 1 (NCBI)	15.8	1.8	10.0	1.64	0.88	1.9
PMED4_13821	gmhA	putative phosphoheptose isomerase (NCBI)	0.2	23.3	23.1	1.30	0.56	2.3
PMED4_13831	rfaE	putative ADP-heptose synthase (NCBI)	0.0	23	22.6	1.34	0.32	4.2
PMED4_13961	NA	conserved hypothetical (NCBI)	4	4.8	0.8	0.94	0.12	7.8
PMED4_13971	NA	methyltransferase (NCBI)	3.9	11.6	7.8	1.06	0.08	13.3
PMED4_13991	adhC	Zinc-containing alcohol dehydrogenase superfamily (N	3.7	13.8	10.1	1.14	0.10	11.4
PMED4_14001	NA	possible N-terminal fragment of transketolase (NCBI)	2.6	16.3	13.7	0.96	0.24	4.0
PMED4_14011	NA	unknown (NCBI)	3.4	5.4	2.0	0.90	0.16	5.6

Locus Tag	Gene Name	Gene Description	Time of Peak Transcript Abundance	Time of Peak Protein Abundance	Protein-Transcript Lag, hours	Transcript Amplitude, log2	Protein Amplitude, log2	Transcript: Protein Amplitude Ratio
PMED4_14041	hetA	ABC-type multidrug transport system ATPase and per	16.5	24	7.0	1.64	0.38	4.3
PMED4_14111	NA	Carbamoyltransferase (NCBI)	3.6	12.9	9.3	1.26	0.12	10.5
PMED4_14121	NA	hypothetical protein (NCBI)	5.4	12.7	7.4	1.04	0.20	5.2
PMED4_14241	NA	pyridoxal-phosphate-dependent aminotransferase (NC	17.3	13.8	20.6	0.88	0.12	7.3
PMED4_14291	argF	Ornithine carbamoyltransferase (Katherine Huang)	3.3	18.0	14.8	1.44	2.04	0.7
PMED4_14301	NA	cell division protein FtsH3 (NCBI)	4.8	11.0	6.1	2.06	0.22	9.4
PMED4_14361	pheS	Phenylalanyl-tRNA synthetase alpha chain (NCBI)	0.8	17.3	16.5	0.56	0.44	1.3
PMED4_14501	phoH	PhoH-like phosphate starvation-inducible protein (NCI	23.5	6.5	7.1	2.30	0.40	5.8
PMED4_14521	ffh	signal recognition particle protein (SRP54) (NCBI)	21	10.6	13.5	0.86	0.46	1.9
PMED4_14531	NA	conserved hypothetical protein (NCBI)	16	21.2	5.2	2.38	0.34	7.0
PMED4_14631	trpC	Indole-3-glycerol phosphate synthase (Katherine Huar	2.0	0.8	22.7	0.70	0.16	4.4
PMED4_14641	lpd	putative dihydrolipoamide dehydrogenase (NCBI)	1.6	21.6	20.1	0.66	0.22	3.0
PMED4_14761	ftsZ	Cell division protein FtsZ:Tubulin/FtsZ family (NCBI)	16.8	19.9	3.1	4.56	0.42	10.9
PMED4_14771	panB	putative Ketopantoate hydroxymethyltransferase (NCE	14.7	14	23.8	1.34	0.18	7.4
PMED4_14801	NA	Clp protease proteolytic subunit (NCBI)	17.7	19.7	2.0	1.24	0.16	7.8
PMED4_14811	NA	Clp protease proteolytic subunit (NCBI)	15.8	20.0	4.2	1.14	0.32	3.6
PMED4_14821	ilvC	Ketol-acid reductoisomerase (NCBI)	0.8	14.6	13.8	0.50	0.10	5.0
PMED4_14891	himA	Bacterial histone-like DNA-binding protein (NCBI)	16.9	20.7	3.8	5.00	0.36	13.9
PMED4_14941	NA	conserved hypothetical protein (NCBI)	22.9	5.1	6.2	1.06	0.34	3.1
PMED4_14981	NA	conserved hypothetical protein (NCBI)	17.4	17.4	0.0	0.46	0.34	1.4
PMED4_15001	pepB	Cytosol aminopeptidase (NCBI)	13.7	22.6	9.0	0.74	0.26	2.8
PMED4_15031	lpxA	UDP-N-acetylglucosamine acyltransferase (NCBI)	22.9	16.4	17.5	0.96	0.36	2.7
PMED4_15041	fabZ	Putative (3R)-hydroxymyristoyl-[acyl carrier protein] de	2.5	7	4.0	1.82	1.22	1.5
PMED4_15061	NA	chloroplast outer envelope membrane protein homolo	20.9	1.8	4.9	1.04	0.08	13.0
PMED4_15091	nblS	two-component sensor histidine kinase (NCBI)	0.0	3	3.1	0.50	0.64	0.8
PMED4_15131	rpmA	50S ribosomal protein L27 (NCBI)	1.9	3.3	1.4	1.84	0.70	2.6
PMED4_15181	NA	Pentapeptide repeats (NCBI)	7.1	13.0	5.9	1.68	0.26	6.5
PMED4_15221	serA	putative D-3-phosphoglycerate dehydrogenase (PGDI	18.0	17.3	23.2	1.90	0.26	7.3
PMED4_16171	NA	conserved hypothetical protein (NCBI)	19	21.0	1.8	2.04	0.24	8.5
PMED4_16411	pyrR	Phosphoribosyl transferase (NCBI)	17.6	6.7	13.1	1.38	1.86	0.7
PMED4_16451	groEL	GroEL protein (Chaperonin cpn60) (NCBI)	21.6	3.9	6.3	1.28	0.16	8.0
PMED4_16461	groES	GroES protein (Chaperonin cpn10) (NCBI)	22.7	23.3	0.6	0.66	0.18	3.7
PMED4_16471	atpD	ATP synthase F1, beta subunit (Katherine Huang)	3.8	9.7	5.9	2.28	0.20	11.4
PMED4_16481	atpC	ATP synthase, Epsilon subunit (NCBI)	4.5	10.1	5.6	2.44	0.70	3.5
PMED4_16601	atpA	ATP synthase F1, alpha subunit (Katherine Huang)	2.7	10.0	7.3	2.16	0.20	10.8

Locus Tag	Gene Name	Gene Description	Time of Peak Transcript Abundance	Time of Peak Protein Abundance	Protein-Transcript Lag, hours	Transcript Amplitude, log2	Protein Amplitude, log2	Transcript: Protein Amplitude Ratio
PMED4_16611	atpH	ATP synthase, delta (OSCP) subunit (NCBI)	2.4	9.1	6.7	2.02	0.26	7.8
PMED4_16631	atpG	ATP synthase B/B' CF(0) (NCBI)	3.1	8.1	5.0	2.52	0.30	8.4
PMED4_16641	atpE	ATP synthase subunit c (Katherine Huang)	3.1	8.1	5.0	2.56	0.48	5.3
PMED4_16721	glnB	Nitrogen regulatory protein P-II (NCBI)	9.2	14.8	5.5	0.82	0.28	2.9
PMED4_16811	BioA	putative diaminopelargonic acid synthase (NCBI)	19.4	1.6	6.1	1.20	0.56	2.1
PMED4_16891	NA	conserved hypothetical protein (NCBI)	13.7	18.3	4.6	0.54	0.24	2.3
PMED4_16931	rpoC2	RNA polymerase beta prime subunit (NCBI)	19.7	22.8	3.1	1.18	0.10	11.8
PMED4_16951	rpoB	RNA polymerase beta subunit (NCBI)	20.2	23.4	3.3	1.34	0.12	11.2
PMED4_16971	rpsT	30s Ribosomal protein S20 (NCBI)	2.9	12.1	9.1	1.70	0.22	7.7
PMED4_17021	nusA	N utilization substance protein A (NCBI)	19	22.8	3.9	1.82	0.16	11.4
PMED4_17101	NA	putative aminotransferase (NCBI)	21.8	22.5	0.6	0.82	0.40	2.1
PMED4_17111	rne	S1 RNA binding domain:Ribonuclease E and G (NCBI)	1.0	4.1	3.0	1.08	0.30	3.6
PMED4_17171	rpsJ	30S ribosomal protein S10 (NCBI)	2.4	3.3	0.9	1.66	0.28	5.9
PMED4_17191	fusA	Elongation factor G (NCBI)	2.4	10.1	7.7	1.52	0.12	12.7
PMED4_17211	rpsL	30S ribosomal protein S12 (NCBI)	2.4	10.0	7.5	1.40	0.96	1.5
PMED4_17221	gltB	Ferredoxin-dependent glutamate synthase, Fd-GOGA	15.8	18	2.4	1.70	0.50	3.4
PMED4_17291	psaL	Photosystem I PsaL protein (subunit XI) (NCBI)	6.5	9	2.1	1.48	0.20	7.4
PMED4_17331	psaB	Photosystem I PsaB protein (NCBI)	9.0	7.7	22.7	0.88	0.46	1.9
PMED4_17341	psaA	Photosystem I PsaA protein (NCBI)	9.5	3.3	17.8	0.92	0.56	1.6
PMED4_17441	rplQ	50S ribosomal protein L17 (NCBI)	23.8	10.4	10.6	1.50	0.34	4.4
PMED4_17451	rpoA	Bacterial RNA polymerase, alpha chain (NCBI)	23.0	22.1	23.1	1.32	0.08	16.5
PMED4_17461	rpsK	30S ribosomal protein S11 (NCBI)	0.8	0.3	23.5	1.36	0.16	8.5
PMED4_17471	rpsM	30S ribosomal protein S13 (NCBI)	2.2	8.9	6.6	1.70	0.28	6.1
PMED4_17491	adk	Adenylate kinase (NCBI)	1.3	21.8	20.5	1.80	0.38	4.7
PMED4_17511	rplO	50S ribosomal protein L15 (NCBI)	1.6	7.6	5.9	1.80	0.30	6.0
PMED4_17521	rpsE	30S ribosomal protein S5 (NCBI)	0.3	1.4	1.1	1.58	0.28	5.6
PMED4_17541	rplF	50S ribosomal protein L6 (NCBI)	0.5	5.6	5.2	1.30	0.16	8.1
PMED4_17551	rpsH	30S ribosomal protein S8 (NCBI)	0.9	11.8	11.0	1.52	0.06	25.3
PMED4_17561	rplE	50S ribosomal protein L5 (NCBI)	0.6	6.9	6.2	1.30	0.36	3.6
PMED4_17591	rpsQ	30S Ribosomal protein S17 (NCBI)	0	10.9	10.6	1.74	0.10	17.4
PMED4_17611	rplP	50S ribosomal protein L16 (NCBI)	1.2	8.5	7.3	1.82	0.40	4.6
PMED4_17631	rplV	50S ribosomal protein L22 (NCBI)	0.9	6.1	5.1	1.90	0.34	5.6
PMED4_17641	rpsS	30S Ribosomal protein S19 (NCBI)	2.5	4.4	2.0	2.62	0.28	9.4
PMED4_17651	rplB	50S ribosomal protein L2 (NCBI)	0.9	7.3	6.4	1.86	1.42	1.3
PMED4_17681	rplC	50S ribosomal protein L3 (NCBI)	0.7	6.3	5.6	2.18	0.46	4.7

Locus Tag	Gene Name	Gene Description	Time of Peak Transcript Abundance	Time of Peak Protein Abundance	Protein-Transcript Lag, hours	Transcript Amplitude, log2	Protein Amplitude, log2	Transcript: Protein Amplitude Ratio
PMED4_17721	recA	RecA bacterial DNA recombination protein (NCBI)	16.6	17.8	1.2	3.10	0.36	8.6
PMED4_17781	NA	conserved hypothetical protein (NCBI)	16	19.7	3.7	3.34	0.36	9.3
PMED4_17941	rnd	putative ribonuclease D (NCBI)	12.3	22.5	10.2	1.22	0.58	2.1
PMED4_18061	icd	Isocitrate dehydrogenase (NCBI)	16.7	19.5	2.8	3.60	0.40	9.0
PMED4_18111	glgP	phosphorylase (NCBI)	16.4	21.1	4.7	4.28	0.56	7.6
PMED4_18171	psaC	Photosystem I subunit PsaC (NCBI)	8.8	16.7	7.9	1.22	0.10	12.2
PMED4_18181	acpP	acyl carrier protein (ACP) (NCBI)	3.9	6.3	2.4	1.26	0.82	1.5
PMED4_18201	tktA	Transketolase (NCBI)	2.8	12.8	10.0	1.70	0.08	21.3
PMED4_18211	thiC	ThiC family (NCBI)	15	18.9	3.5	4.14	0.84	4.9
PMED4_18281	lysS	Lysyl-tRNA synthetase (NCBI)	20.5	20	23.8	1.54	0.24	6.4
PMED4_18291	NA	two-component response regulator (NCBI)	3	10.0	7.3	0.60	0.18	3.3
PMED4_18321	mreB	Rod shape determining protein (NCBI)	16.3	19.8	3.5	1.66	0.30	5.5
PMED4_18331	ssb	single-stranded DNA-binding protein (NCBI)	16.1	0.7	8.7	4.22	0.86	4.9
PMED4_18351	sam1	putative adenosylhomocysteinase (NCBI)	11.3	16.9	5.7	1.02	0.20	5.1
PMED4_18401	mgtE	MgtE family, putative magnesium transport protein (N	17.0	2.2	9.1	0.64	0.18	3.6
PMED4_18441	gyrB	DNA gyrase, subunit B (NCBI)	15.6	21.0	5.4	1.26	0.24	5.3
PMED4_18491	secA	Preprotein translocase SecA subunit (NCBI)	3	5.4	2.4	0.72	0.08	9.0
PMED4_18601	NA	conserved hypothetical protein (NCBI)	13.8	16.8	3.0	2.02	0.42	4.8
PMED4_18621	NA	Predicted hydrolase of the metallo-beta-lactamase su	20.0	1.6	5.6	0.84	0.20	4.2
PMED4_18641	asd	aspartate Semialdehyde dehydrogenase (NCBI)	1.4	3.5	2.0	0.96	0.20	4.8
PMED4_18651	tig	FKBP-type peptidyl-prolyl cis-trans isomerase (PPIase	1.2	5.5	4.3	1.18	0.20	5.9
PMED4_18661	clpP2	Clp protease proteolytic subunit (NCBI)	15.9	18.9	3.0	1.08	0.26	4.2
PMED4_18671	clpX	Clp protease ATP-binding subunit, ClpX (NCBI)	16.4	20.4	4.0	3.42	0.96	3.6
PMED4_18721	rplT	50S ribosomal protein L20 (NCBI)	0.9	0.1	23.2	1.30	0.38	3.4
PMED4_18751	sqdB	sulfolipid (UDP-sulfoquinovose) biosynthesis protein (N	5.4	8.4	3.0	1.06	0.08	13.3
PMED4_18841	dnaB	DnaB replicative helicase (NCBI)	18.5	16	21.8	0.98	0.46	2.1
PMED4_18991	aspS	Aspartyl-tRNA synthetase (NCBI)	1.7	4.4	2.8	0.98	0.14	7.0
PMED4_19161	aroE	Shikimate / quinate 5-dehydrogenase (NCBI)	17.0	16.6	23.6	1.28	1.40	0.9
PMED4_19181	argG	Argininosuccinate synthase (NCBI)	18.2	1.5	7.3	1.16	0.56	2.1
PMED4_19241	uvrA	Excinuclease ABC, A subunit, ATP/GTP-binding site m	12.1	18.3	6.2	0.86	0.22	3.9
PMED4_19261	NA	possible protein kinase:ABC1 family (NCBI)	21	12.4	15.7	0.66	0.52	1.3
PMED4_19281	thrC	Threonine synthase: Pyridoxal-5'-phosphate-depende	23.8	22.6	22.7	0.60	0.16	3.8
