# Supplementary material for: Transcriptome and Proteome Dynamics of a Light-Dark Synchronized Bacterial Cell Cycle
Source: PLoS One. 2012 Aug 29;7(8):e43432. doi: 10.1371/journal.pone.0043432 (PMC3430701; doi:10.1371/journal.pone.0043432)
Supplement: Table S3 — Cycling parameters of genes of the Calvin cycle (green), pentose phosphate pathway (red) and shared between the two (blue). (PDF) [file pone.0043432.s014.pdf]

Locus Tag	Gene Name	Gene Description	Time of Peak Transcript Abundance	Time of Peak Protein Abundance	Protein-Transcript Lag, hours	Transcript Amplitude, log2	Protein Amplitude, log2	Transcript: Protein Amplitude Ratio
PMED4_08711	prkB	phosphoribulokinase (NCBI)	5	12.2	7.6	2.10	0.14	15.0
PMED4_05991	rbcL	Ribulose bisphosphate carboxylase, large chain (NCBI)	4.3	13	9.1	5.20	0.32	16.3
PMED4_06001	rbcS	Ribulose bisphosphate carboxylase, small chain (NCBI)	4	12.8	8.4	4.82	0.40	12.1
PMED4_02011	pgk	Phosphoglycerate kinase (NCBI)	7.0	15.1	8.1	2.94	0.42	7.0
PMED4_00221	gap2	Glyceraldehyde 3-phosphate dehydrogenase(NADP+)(phosphorylating) (NCBI)	4.6	13.5	8.9	1.56	0.18	8.7
PMED4_09231	tpi	Triosephosphate isomerase (NCBI)	5.3	6.3	1.1	1.78	0.44	4.0
PMED4_08671	cbbA	Fructose-bisphosphate/sedoheptulose-1, 7-bisphosph ate aldolase (NCBI)	3.3	10.1	6.9	2.50	0.52	4.8
PMED4_08521	glpX	Fructose-1,6-bisphosphatase/sedoheptulose-1, 7-bis phosphatase (NCBI)	4.4	12.3	7.9	2.10	0.10	21.0
PMED4_18201	tktA	Transketolase (NCBI)	2.8	12.8	10.0	1.70	0.08	21.3
PMED4_08511	rpe	Ribulose-phosphate 3-epimerase (NCBI)	3.1	4.2	1.2	2.44	0.32	7.6
PMED4_05681	tal	Transaldolase (NCBI)	15.8	21	5.6	3.88	1.02	3.8
PMED4_10011	pgi	Phosphoglucose isomerase (PGI) (NCBI)	13.3	18.8	5.5	1.10	0.24	4.6
PMED4_12201	zwf	Glucose-6-phosphate dehydrogenase (NCBI)	17.2	21.3	4.1	2.38	0.42	5.7
PMED4_08551	gnd	6-phosphogluconate dehydrogenase (NCBI)	16.6	21.5	5.0	3.26	0.32	10.2
