# Supplementary material for: Transcriptome and Proteome Dynamics of a Light-Dark Synchronized Bacterial Cell Cycle
Source: PLoS One. 2012 Aug 29;7(8):e43432. doi: 10.1371/journal.pone.0043432 (PMC3430701; doi:10.1371/journal.pone.0043432)
Supplement: Table S4 — Proportion of genes with detected sense or anti-sense transcript at each experimental timepoint. (PDF) [file pone.0043432.s015.pdf]

	Time point (hour)													
	**0**	**2**	**4**	**6**	**8**	**10**	**12**	**14**	**16**	**18**	**20**	**22**	**24**	**26**
**Proportion of genes with sense transcript detected (%)**	97.8	97.5	97.2	97.1	96.5	96.7	98.3	96.0	95.0	95.6	95.7	96.3	96.3	96.6
**Proportion of genes with anti-sense transcript detected (%)**	36.2	41.2	41.6	44.0	31.5	38.2	40.6	31.5	33.4	28.5	32.6	34.8	36.8	24.9
