# Supplementary material for: Transcriptome and Proteome Dynamics of a Light-Dark Synchronized Bacterial Cell Cycle
Source: PLoS One. 2012 Aug 29;7(8):e43432. doi: 10.1371/journal.pone.0043432 (PMC3430701; doi:10.1371/journal.pone.0043432)
Supplement: Table S5 — Effect of data-quality filtering steps (see Methods) on proteomics dataset size. (PDF) [file pone.0043432.s016.pdf]

Filtering Step	Unique LC-MS $^{14}N/^{15}N$ peakgroup pairs	Protein timepoints	Detected & quantified proteins	Proteins detected at all 14 timepoints
Full diel dataset	95,542	8,812	1,021	360
Peak CV	76,434	8,181	967	307
Peaks per timepoint	73,185	6,157	548	170
IIR peak outliers	66,661	6,157	548	170
Timepoint outliers	66,186	6,087	548	154
